# Combined inhibition of ribonucleotide reductase and WEE1 induces synergistic anticancer activity in Ewing’s sarcoma cells

**DOI:** 10.1186/s12885-025-13691-2

**Published:** 2025-02-17

**Authors:** Judy Ziener, Julián Andrés Henao-Restrepo, Johanna Leonhardi, Max-Johann Sturm, Sabine Becker, Diana M. Morales-Prieto, Till Milde, James F. Beck, Jürgen Sonnemann

**Affiliations:** 1 Department of Paediatric and Adolescent Medicine, Jena University Hospital, Friedrich Schiller University Jena, Jena, Germany; 2 Research Centre Lobeda, Jena University Hospital, Friedrich Schiller University Jena, Jena, Germany; 3 Placenta Lab, Department of Obstetrics, Jena University Hospital, Jena, Germany; 4 Comprehensive Cancer Centre Central Germany (CCCG), Jena, Germany; 5 Hopp Children’s Cancer Center Heidelberg (KiTZ), Heidelberg, Germany; 6 Clinical Cooperation Unit Pediatric Oncology, German Cancer Research Center Heidelberg (DKFZ), Heidelberg, Germany; 7 Klinik für Kinder- und Jugendmedizin, Friedrich-Schiller-Universität Jena, Am Klinikum 1, D-07747 Jena, Germany

**Keywords:** Ewing’s sarcoma, Targeted therapy, Ribonucleotide reductase, WEE1, PARP, Triapine, Adavosertib, ZN-c3, Olaparib, Veliparib

## Abstract

**Background:**

Ewing’s sarcoma is a childhood bone and soft tissue cancer with poor prognosis. Treatment outcomes for Ewing’s sarcoma patients have improved only modestly over the past decades, making the development of new treatment strategies paramount. In this study, the combined targeting of ribonucleotide reductase (RNR) and WEE1 was explored for its effectiveness against Ewing’s sarcoma cells.

**Methods:**

The RNR inhibitor triapine and the WEE1 inhibitors adavosertib and ZN-c3 were tested in p53 wild-type and p53 mutant Ewing’s sarcoma cells. The combination of adavosertib with the PARP inhibitors olaparib and veliparib was tested for comparison. Combinatorial effects were determined by flow cytometric analyses of cell death, loss of mitochondrial membrane potential and DNA fragmentation as well as by caspase 3/7 activity assay, immunoblotting and real-time RT-PCR. The drug interactions were assessed using combination index analysis.

**Results:**

RNR and WEE1 inhibitors were weakly to moderately effective on their own, but highly effective in combination. The combination treatments were similarly effective in p53 wild-type and p53 mutant cells. They synergistically induced cell death and cooperated to elicit mitochondrial membrane potential decay, to activate caspase 3/7 and to trigger DNA fragmentation, evidencing the induction of the apoptotic cell death cascade. They also cooperated to boost CHK1 phosphorylation, indicating augmented replication stress after combination treatment. In comparison, the combination of adavosertib with PARP inhibitors produced weaker synergistic effects.

**Conclusion:**

Our findings show that combined inhibition of RNR and WEE1 was effective against Ewing’s sarcoma in vitro. They thus provide a rationale for the evaluation of the potential of combined targeting of RNR and WEE1 in Ewing’s sarcoma in vivo.

**Supplementary Information:**

The online version contains supplementary material available at 10.1186/s12885-025-13691-2.

## Background

Ewing’s sarcoma (ES) is a highly malignant tumour of bone and soft tissue that occurs primarily in childhood and adolescence [1, 2]. The standard of care therapy of ES consists of intensive neoadjuvant induction chemotherapy, followed by surgery and/or radiation and adjuvant consolidation chemotherapy [3]. A plethora of consecutive studies since the 1960s led to a remarkable progress in the treatment of ES: the 5-year survival rate for localised disease increased from less than 10% before the introduction of chemotherapy to ∼ 75% today [4]. However, the prognosis for patients with primary disseminated disease or relapse remains poor, with a 5-year survival rate of less than 40% [2]. Most noteworthy, only minimal gains in treatment efficacy with regard to response and survival of ES patients have been made since the turn of the century, indicating that the optimisation of cytotoxic chemotherapy dosing and combinations has reached its limits [5]. New therapeutic approaches, therefore, are needed to further improve the outcome of patients with ES [3, 6, 7].

ES is characterised by a balanced chromosome translocation resulting in a fusion oncogene. The most frequent variant, accounting for 85% of cases, is the translocation t(11;22)(q24;q12), which leads to the fusion of the Ewing’s sarcoma breakpoint region 1 (*EWSR1*) gene of the FET family with the Friend leukaemia virus integration 1 (*FLT1*) gene of the ETS family [8]. This translocation gives rise to the fusion oncoprotein EWS::FLI1, which acts as a neomorphic transcription factor by regulating the expression of genes involved in, e.g., cell proliferation, cell migration, cell cycle control, cell death and signal transduction [1, 9]. EWS::FLI1 hence is a natural candidate target for ES. Direct EWS::FLI1 targeting, however, is complicated by its function as a transcription factor [6, 10].

Yet EWS::FLI1 could be exploited indirectly by targeting its downstream mediators [6, 10]. For instance, EWS::FLI1 promotes R-loop formation and blocks BRCA function– conferring a ‘BRCAness’ phenotype [11]– leading to high levels of endogenous replication stress in ES cells [12]. ES is thus supposed to be particularly sensitive to agents that target the replication stress response (RSR) [13–16]. The ATR/CHK1/WEE1 signalling cascade is a pivotal mediator of RSR and, as such, a promising anticancer target [17–19]. In line with this conception, preclinical studies have already demonstrated single-agent activity of ATR, CHK1 and WEE1 inhibitors in ES [20–22]. Furthermore, RSR targeting is an appealing approach, as RSR is more important for the proliferation and survival of cancer cells than for normal cells, which offers the prospect of limiting medication-related side effects [17].

However, RSR-targeted drugs are rarely persistently effective as monotherapy in the majority of tumours [23]. Moreover, single-agent treatments are generally insufficient to achieve long-lasting remission in ES patients [3]. Since the development of drug resistance may be overcome by combination treatment, we recently set out to identify effective combination partners for RSR-targeted inhibitors as a therapeutic strategy for ES. We have shown that the ATR inhibitor (ATRi) VE821 effectively cooperated with the HSP90 inhibitor AUY922 [24] as well as the ribonucleotide reductase (RNR) inhibitors (RNRi) triapine (also known as 3-AP) and didox [25] in killing ES cells.

RNRi hold particular promise for enhancing the effectiveness of RSR-targeted agents [18]. RNR catalyses the rate-determining step in the synthesis of deoxyribonucleotides (dNTPs) [26]. The upregulation of the RNR subunit M2 (RRM2; also known as β2) increases the supply of dNTPs, fostering the recovery from replication stress, thus counteracting the effects of RSR-targeted therapeutics [27, 28]. RRM2 overexpression was reported to be associated with poor overall survival in ES patients [29], and RRM2 was identified as a potential treatment target in ES [30]. However, although initially responsive to the RRM2 inhibitor triapine, ES cells were found to develop relative resistance to the drug over time, suggesting that RNRi monotherapy will not be sufficient for durable disease control [29].

Single-agent therapy with either RSR-targeted drugs or RNRi is therefore very likely to be inadequate for the treatment of ES, but their combination holds promise. In a former study, we demonstrated that combined ATR and RNR inhibition produced a synergistic antineoplastic effect in ES cells [25]. In the present one, we have extended the exploration into the feasibility of combined RSR and RNR inhibition to the combination of triapine with inhibitors of the ATR downstream kinase WEE1, a promising therapeutic target in itself [31, 32]. In both studies, we used the RNRi triapine because it has been shown to be effective against cancer cells that are resistant to the widely applied RNRi hydroxyurea [33] and because it is one of the most commonly used RNRi in clinical trials [34–37]. We show here that triapine synergised with the WEE1 inhibitors (WEE1i) adavosertib (also known as AZD1775 and MK-1775) and ZN-c3 (also known as azenosertib) in exerting anticancer action on ES cells, pointing to the usefulness of this drug combination in the therapy of ES.

## Methods

### Cell culture

WE-68 (RRID: CVCL_9717) cells were kindly provided by Dr F. van Valen (Münster, Germany), SK-ES-1 cells (RRID: CVCL_0627) and A673 cells (RRID: CVCL_0080) were purchased from the DSMZ (Braunschweig, Germany) and Sigma Aldrich (Deisenhofen, Germany), respectively. RPMI 1640 medium (Capricorn Scientific, Ebsdorfergrund, Germany) was used to culture WE-68 and SK-ES-1 cells, and DMEM (Lonza, Basel, Switzerland) was used to culture A673 cells. Media were supplemented with 10% foetal bovine serum (Capricorn Scientific), 100 units/ml penicillin G sodium and 100 µg/ml streptomycin sulphate (Lonza). Cells were cultured in rat-tail collagen-coated (5 µg/cm^2^; Merck, Darmstadt, Germany) cell culture vessels. Cells were maintained in an incubator at 37 °C and 5% CO_2_ and passaged at approximately 90% confluence. Mycoplasma contamination was ruled out with the qPCR Mycoplasma Testkit from AppliChem (Darmstadt, Germany).

### Treatment of cells

WE-68 and SK-ES-1 cells were cultured in 24-well tissue culture plates; cells were seeded at 75,000 cells/well for flow-cytometric analyses and at 100,000 cells/well for caspase 3/7 activity measurements. A673 cells were cultured in 6-well tissue culture plates; cells were seeded at 100,000 cells/well for flow-cytometric analyses and at 150,000 cells/well for caspase 3/7 activity measurements. For PCR analyses, cells were cultured in 6-well tissue culture plates, WE-68 and SK-ES-1 cells at 400,000 cells/well and A673 cells at 150,000 cells/well. For immunoblotting, cells were seeded in 25 cm^2^ tissue culture flasks at 10^6^ cells/flask. Twenty-four hours after seeding, cells were treated with triapine (0.125–1 µM; Selleck Chemicals, Planegg, Germany), adavosertib (0.05–0.5 µM; Selleck Chemicals), ZN-c3 (0.3–0.5 µM; Selleck Chemicals), olaparib (0.1–2 µM; Biomol, Hamburg, Germany) and/or veliparib (2.5–20 µM; Biomol) and incubated for 24 h (caspase 3/7 activity, immunoblotting, PCR) or 48 h (flow-cytometric analyses). In the respective experiments, cells were pretreated with the pan-caspase inhibitor z-VAD-fmk (20 µM; Enzo Life Sciences, Lörrach, Germany) 1 h before treatment with triapine, adavosertib and/or ZN-c3.

### Flow-cytometric analysis of cell death, loss of mitochondrial transmembrane potential (Δ*ψ*_m_) and DNA fragmentation

Cell death was determined by flow-cytometric analysis using propidium iodide (PI; Sigma Aldrich) to assess cell membrane integrity. After harvesting, cells were incubated in 2 µg/ml PI in PBS at 4 °C for 5 min in the dark. Loss of Δ*ψ*_m_ was determined by flow-cytometric analysis using 3,3’-dihexyloxacarbocyanine iodide (DiOC_6_(3); Thermo Fisher Scientific, Dreieich, Germany). Cells were incubated with 50 nM DiOC_6_(3) at 37 °C for 45 min in the dark prior to harvesting. DNA fragmentation was determined by assessing cells for PI incorporation into DNA. After harvesting, cells were washed twice with PBS and fixed in 70% ethanol at − 20 °C overnight. After washing, cells were resuspended in PBS containing 1% glucose, 2.5 µl/ml ribonuclease A (Roche, Mannheim, Germany) and 50 µg/ml PI and incubated at 4 °C for 45 min in the dark. 10,000 cells (cell death and Δ*ψ*_m_ loss) or 20,000 cells (DNA fragmentation) per sample were analysed on a BD FACS Canto II (Heidelberg, Germany) with BD FACSDiva software. Gates were placed to exclude debris and aggregates.

To assess the combination treatments for synergistic or antagonistic effects, the results of the cell death determinations were analysed using the combination index (CI) method according to Chou and Talalay [38] with Calcusyn software from Biosoft (Cambridge, UK). Theoretically (i.e., under ideal conditions), CI values of < 1, = 1 and > 1 indicate synergistic, additive and antagonistic effects, respectively. Here, only CI values < 0.8 were considered synergistic.

### Caspase 3/7 activity

Caspase 3/7 activity was measured using the caspase 3/7 substrate acetyl-Asp-Glu-Val-Asp-amido-4-methyl-coumarin (Ac-DEVD-AMC, Bachem, Weil am Rhein, Germany). After harvesting, cells were lysed in 10 mM NaH_2_PO_4_/NaHPO_4_, 10 mM Tris-HCL (pH 7.5), 130 mM NaCl, 10 mM Na_4_P_2_O_7_, 1% Triton X-100 at 4 °C for 15 min in the dark. Samples were mixed with 20 mM Hepes (pH 7.5), 10% glycerol, 2 mM DTT and 25 µg/ml Ac-DEVD-AMC. The fluorescence of the released AMC was detected on a Tecan Infinite M200 Pro (Crailsheim, Germany) plate reader using an excitation wavelength of 355 nm and an emission wavelength of 440 nm. Relative caspase 3/7 activities are provided as the ratio of the emission of treated to untreated cells.

### Immunoblotting

Cells were centrifuged at 250 x g for 5 min and lysed in 200 µl RIPA buffer (Abcam, Cambridge, UK) supplemented with 20 µl/ml protease and phosphatase inhibitor cocktails (Serva Electrophoresis, Heidelberg, Germany). 30 µg of protein per sample were prepared in Laemmli SDS sample buffer (Thermo Fisher Scientific) and incubated at 85 °C for 3 min. After standard SDS-PAGE using 4–12% precast gels (Serva), proteins were electrophoretically transferred to PVDF membranes (Thermo Fisher Scientific). After blocking for 1 h in TBS (pH 7.6) containing 5% BSA and 0.1% Tween-20, the membranes were incubated overnight at 4 °C with the following antibodies: anti-CHK1 (1:300; Cell Signaling Technology (CST), Leiden, Netherlands, #2360, RRID: AB_2080320), anti-phospho-Ser345-CHK1 (1:300; CST, #2348, RRID: AB_331212) and anti-p53 (1:300; Santa Cruz Biotechnology, Heidelberg, Germany, #sc-126, RRID: AB_628082). Equal loading of protein was verified by the detection of GAPDH (1:3000; CST, #2118, RRID: AB_561053). HRP-conjugated anti-mouse IgG (1:3000; CST, #7076, RRID: AB_330924) and HRP-conjugated anti-rabbit IgG (1:3000; CST, #7074, RRID: AB_2099233) were used as secondary antibodies followed by detection of specific signals using Immobilon Forte Western HRP Substrate (Sigma Aldrich). Imaging was done on an MF ChemiBis 3.2 imaging system (DNR Bio Imaging Systems, Neve Yamin, Israel).

### Real-time RT-PCR

Procedures were done in accordance with the manufacturers’ instructions. RNA was isolated using Peqgold Total RNA Kit including DNase digestion (VWR International, Dresden, Germany) and reverse-transcribed into cDNA using the Omniscript RT Kit (Qiagen GmbH, Hilden, Germany). Real-time PCR was performed on an Applied Biosystems 7900HT Real-Time PCR System (Thermo Fisher Scientific). Reactions were carried out as duplicates using Applied Biosystems Gene Expression Assays and TaqMan Universal PCR Master Mix. Gene expressions of *CDKN1A* (ID: Hs00355782_m1), *BBC3* (ID: Hs00248075_m1) and *TP53* (Hs01034249_m1) were normalised to *B2M* (ID: Hs00187842_m1) gene expression levels. Data analysis was done with SDS2.4 software (Applied Biosytems). The relative gene expression levels were calculated with the 2(^–ΔΔCt^) method.

### Statistical analysis

Results presented are the mean ± SEM of each three independent experiments. A heteroscedastic, two-tailed Student’s *t* test was used for statistical analysis using Microsoft Excel (*p < 0.05, **p < 0.01, ***p < 0.001). Additional statistical analysis (shown in Tables S11–S16) was done by Kruskal-Wallis test followed by Dunn’s test using RStudio version 4.3.0.

## Results

### Combination treatment of triapine with WEE1i synergises in the induction of cell death in ES cells

**Fig. 1:**
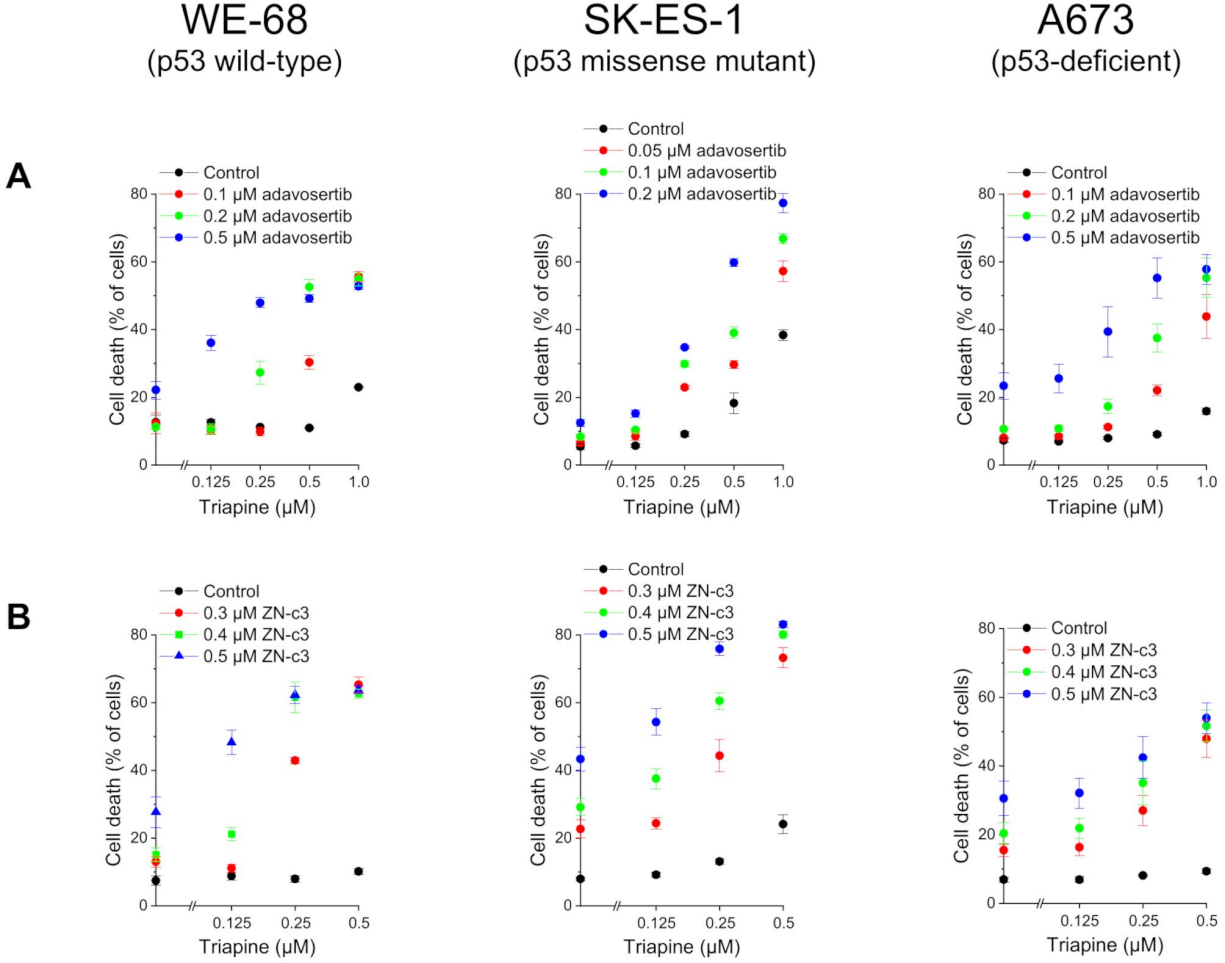
RNRi and WEE1i cooperate in inducing cell death in ES cells. Cells were exposed to triapine in combination with (**A**) adavosertib or (**B**) ZN-c3 for 48 h. Cell death was determined by flow-cytometric analysis of PI uptake. Means ± SEM of each three independent measurements are shown

**Fig. 2:**
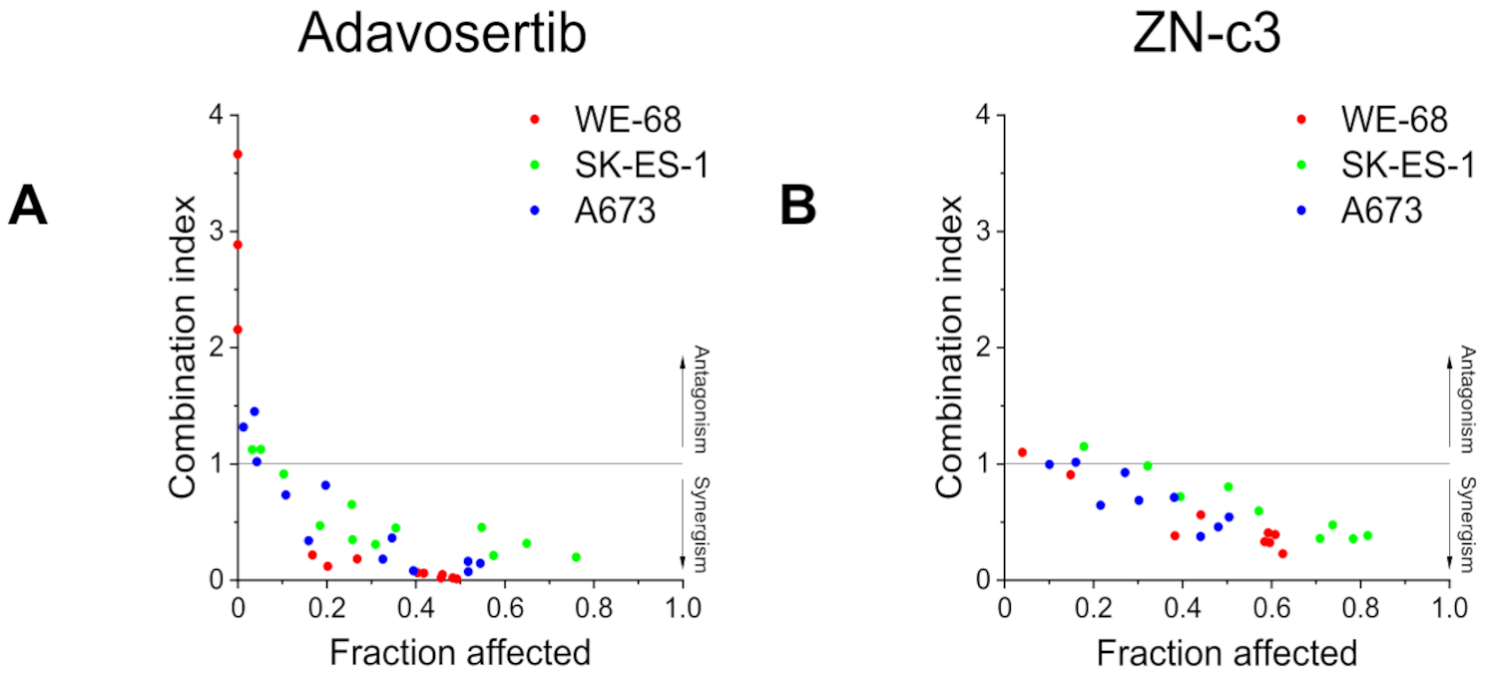
CI values for triapine plus adavosertib or ZN-c3 in ES cells. Based on data from (**A**) Fig. 1A and (**B**) Fig. 1B, CI values were calculated with the Chou-Talalay method

To assess a possible favourable antineoplastic interaction of RNR and WEE1i in ES, we initially determined cell death by flow cytometric analysis of PI uptake. We used three ES cell lines with different p53 status, namely, p53 wild-type WE-68 cells, p53 homozygous missense mutant (C176F) SK-ES-1 cells [39] and p53-deficient A673 cells [40], to address a potential impact of p53 on the combination effect. Cells were treated with the RNRi triapine and the WEE1i adavosertib for 48 h. In WE-68 and A673 cells, single treatment with either agent elicited maximally 23.4 ± 3.9% cell death in the concentration range investigated, while triapine-adavosertib combination treatment resulted in up to 57.8 ± 4.5% cell death (Fig. 1A). SK-ES-1 cells were more sensitive to triapine, i.e., triapine single treatment induced up to 38.4 ± 1.5% cell death. Yet in combination with adavosertib, which was marginally cytotoxic on its own, triapine-induced cell death amounted up to 77.4 ± 2.8% (because of this strong combination effect in SK-ES-1 cells, we used slightly lower concentrations of adavosertib in this cell line). To test the combination effects for synergism, we analysed the data using the CI method [38]. In WE-68 cells, triapine-adavosertib combination treatment produced strong synergistic effects, except for the combinations with 0.125 µM triapine, and for 0.25 µM triapine with 0.1 µM adavosertib (Fig. 2a; numerical values are given in Table S1). In SK-ES-1 cells, synergism was seen in all treatment combinations but those including 0.125 µM triapine (Fig. 2a; numerical values are given in Table S2). In A673 cells, the CI analysis demonstrated a synergistic interplay between triapine and adavosertib, except for the combinations of 0.125 µM triapine with 0.1 and 0.2 µM adavosertib, and for the combination of 0.25 µM triapine with 0.1 µM adavosertib (Fig. 2a; numerical values are given in Table S3).

To test for a potential class effect of WEE1i in ES cells, we examined another WEE1i, ZN-c3. The combination of triapine with ZN-c3 induced equivalent cell death as the combination with adavosertib (Fig. 1B; compare Fig. 1A). Single treatments with ZN-c3 resulted in moderate cell death, with a maximum of 43.3 ± 3.5% in SK-ES-1 cells. Triapine-ZN-c3 combination treatment-triggered cell death reached 65.3 ± 2.3% in WE-68 cells, 83.1 ± 1.0% in SK-ES-1 cells and 53.9 ± 4.4% in A673 cells. The CI analysis demonstrated synergism for this RNR-WEE1i combination, too (Fig. 2B; numerical values are given in Tables S4 to S6). Synergistic effects were observed for all triapine-ZN-c3 combinations in the three cell lines, with the exception of most of the combinations with 0.125 µM triapine.

### Combination treatment of triapine with WEE1i induces apoptosis

**Fig. 3:**
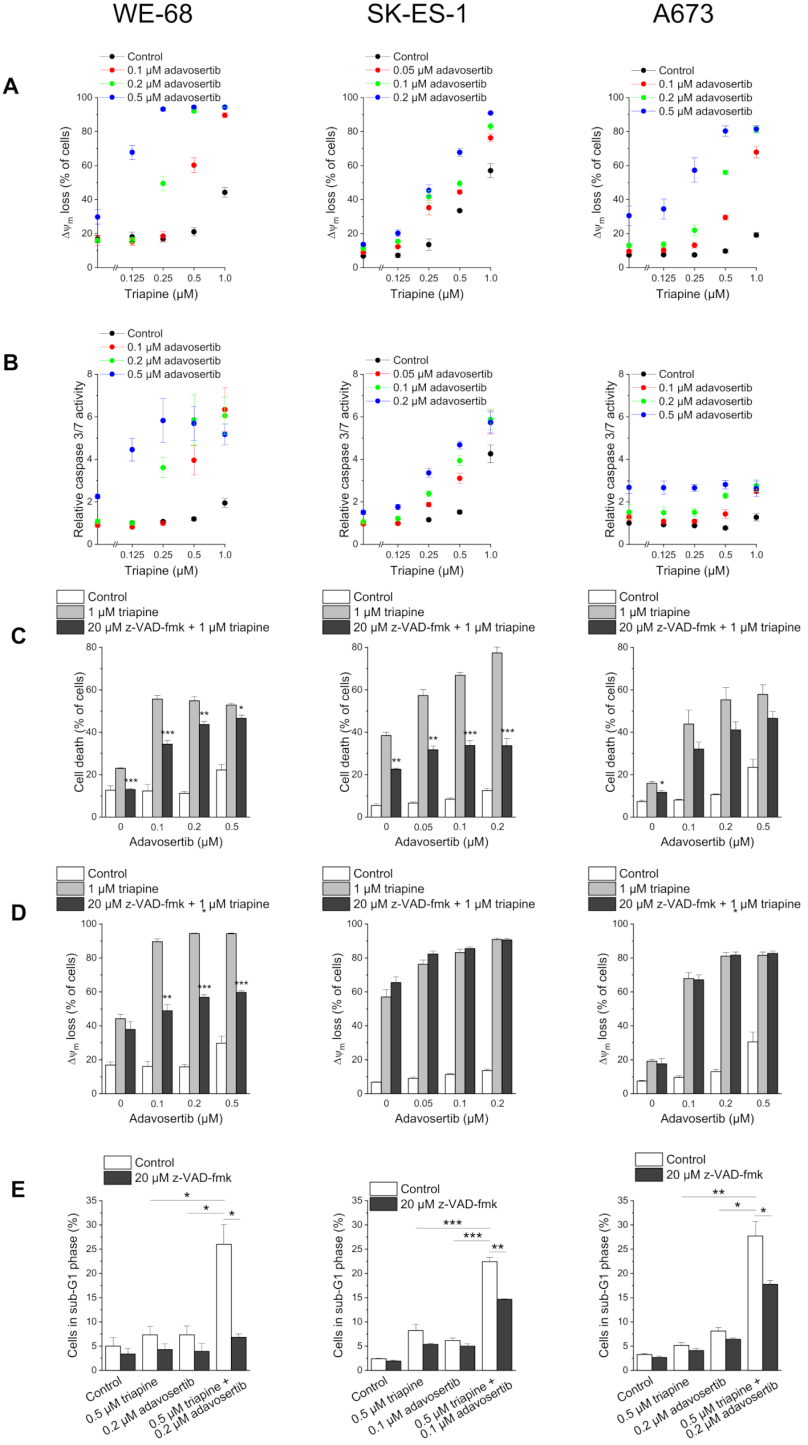
Triapine and adavosertib cooperate in inducing apoptosis in ES cells. Cells were exposed to drugs for (**B**) 24 h or (**A**, **C**, **D**, **E**) 48 h. (**C**, **D**, **E**) z-VAD-fmk was applied 1 h before treatment with triapine-adavosertib. (**A**, **D**) Loss of Δ*ψ*_m_ was determined by flow-cytometric analysis of DiOC_6_(3) staining. (**B**) Caspase 3/7 activity was determined using the fluorogenic substrate Ac-DEVD-AMC; relative caspase 3/7 activities are the ratio of treated cells to untreated cells. (**C**) Cell death was determined by flow-cytometric analysis of PI uptake. (**E**) sub-G1 cells were determined by flow-cytometric analysis of PI-stained ethanol-fixed cells. Means ± SEM of each three independent measurements are shown (*p < 0.05, **p < 0.01, ***p < 0.001; (**C**, **D**) black bars vs. grey bars)

To gain insight into the mode of RNRi-WEE1i-induced cell death, we analysed the effects of the combination treatment by a number of read-outs. Since most cell death pathways including apoptosis involve mitochondria [41], we first studied the action of the triapine-adavosertib combination on Δ*ψ*_m_ dissipation by flow-cytometric analysis of DiOC_6_(3) staining. In keeping with the findings of the cell death determinations, triapine and adavosertib applied as single agents elicited weak to moderate effects in WE-68 and A673 cells, whereas their combination induced Δ*ψ*_m_ decay in up to 94.3 ± 0.4% of WE-68 cells and up to 81.6 ± 2.0% of A673 cells (Fig. 3A). In SK-ES-1 cells, triapine single treatment was again more effective, leading to 57 ± 4.1% Δ*ψ*_m_ loss at its highest concentration. In conjunction with adavosertib, however, the effect was increased to 90.9 ± 0.7%. To test for the involvement of caspases as a second indicator for apoptosis, we assessed caspase 3/7 activity after a 24-h treatment. In WE-68 and SK-ES-1 cells, the results of this assay matched those of the cell death and Δ*ψ*_m_ decay determinations: single treatments with triapine or adavosertib induced some caspase 3/7 activation, while the combination treatment produced potentiated effects (Fig. 3B). However, in A673 cells, the treatments activated caspase 3/7 to a much lesser extent, i.e., triapine induced weak caspase 3/7 activity only in combination with 0.1 µM or 0.2 µM adavosertib.

To evaluate whether caspase 3/7 activation was not only a side effect but essential for triapine-adavosertib-elicited cell death, we used the broad-spectrum caspase inhibitor z-VAD-fmk. As demonstrated in Fig. 3C, z-VAD-fmk impinged on triapine-adavosertib-triggered cell death in WE-68 and SK-ES-1 cells, but had little effect in A673 cells, consistent with the weak caspase 3/7 activation in these cells. The pan-caspase inhibitor also markedly alleviated triapine-adavosertib-mediated Δ*ψ*_m_ decay in WE-68 cells, but not in the other two cell lines (Fig. 3D). To further substantiate the apoptosis-inducing action of triapine-adavosertib combination treatment, we assessed DNA fragmentation by flow-cytometric determination of the sub-G1 fraction of cells with DNA < 2*n*. Figure 3E shows that triapine-adavosertib provoked DNA fragmentation in the three cell lines. It also shows that z-VAD-fmk completely prevented DNA fragmentation in WE-68 cells and partially in SK-ES-1 and A673 cells.

**Fig. 4:**
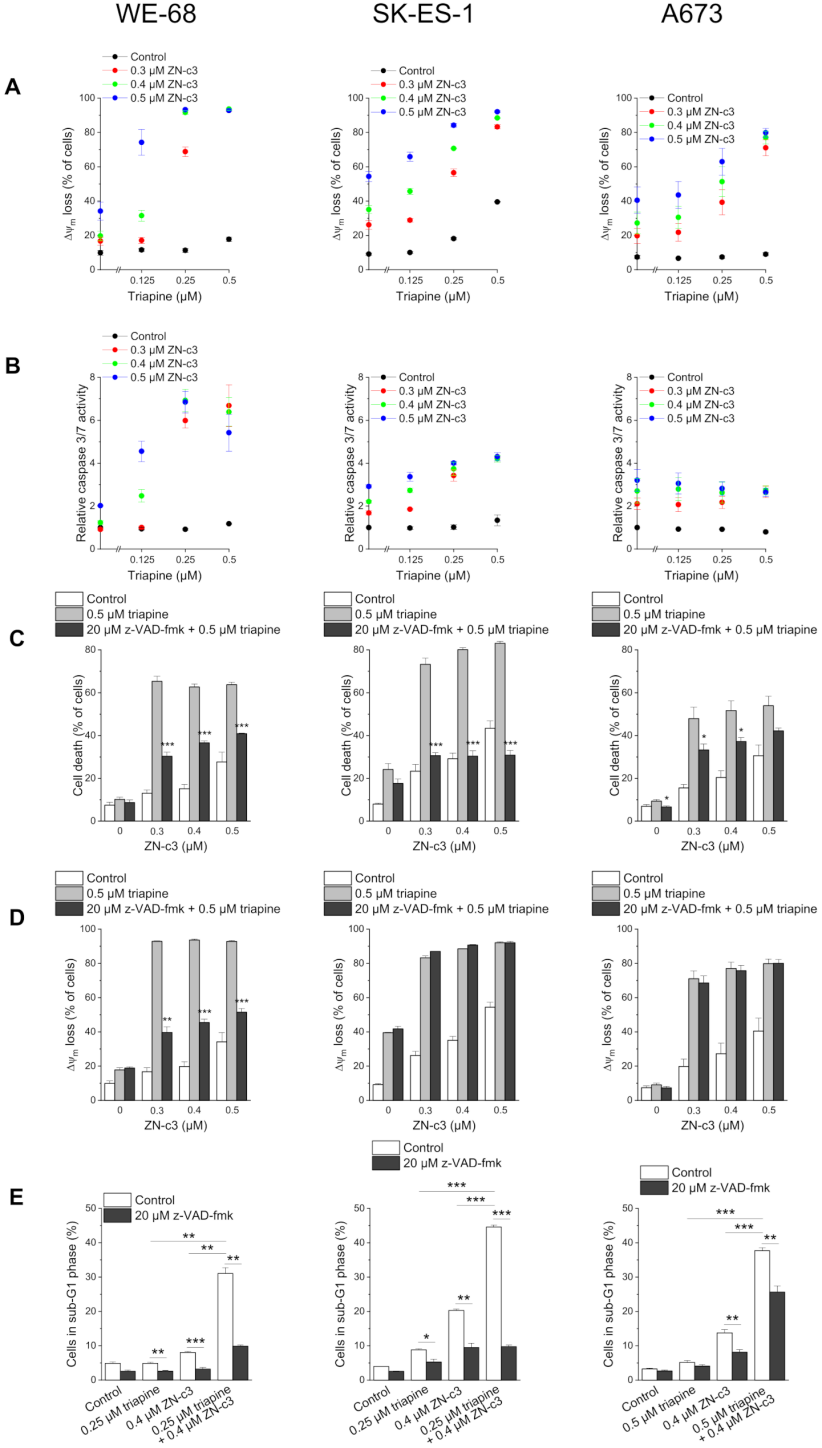
Triapine and ZN-c3 cooperate in inducing apoptosis in ES cells. Cells were exposed to drugs for (**B**) 24 h or (**A**, **C**, **D**, **E**) 48 h. (**C**, **D**, **E**) z-VAD-fmk was applied 1 h before treatment with triapine-ZN-c3. (**A**, **D**) Loss of Δ*ψ*_m_ was determined by flow-cytometric analysis of DiOC_6_(3) staining. (**B**) Caspase 3/7 activity was determined using the fluorogenic substrate Ac-DEVD-AMC; relative caspase 3/7 activities are the ratio of treated cells to untreated cells. (**C**) Cell death was determined by flow-cytometric analysis of PI uptake. (**E**) sub-G1 cells were determined by flow-cytometric analysis of PI-stained ethanol-fixed cells. Means ± SEM of each three independent measurements are shown (*p < 0.05, **p < 0.01, ***p < 0.001; (**C**, **D**) black bars vs. grey bars)

We further examined whether also the combination of triapine with ZN-c3 triggered the apoptotic pathway of cell death. As judged by determining Δ*ψ*_m_ dissipation and caspase 3/7 activity, the combination of triapine with ZN-c3 produced a similar outcome as the combination of triapine with adavosertib (Fig. 4A, B; compare Fig. 3A, B). The pan-caspase inhibitor had a similar impact on triapine-ZN-c3-induced cell death and Δ*ψ*_m_ loss as on the triapine-adavosertib-induced ones as well (Fig. 4C, D; compare Fig. 3C, D). Likewise, the two triapine-WEE1i combinations produced equivalent effects with regard to DNA fragmentation (Fig. 4E; compare Fig. 3E).

### The combination of adavosertib with PARP inhibitors produces less pronounced synergistic effects against ES cells

For comparison with the combination of WEE1i and triapine, we also tested the combination of WEE1i and poly(ADP-ribose)-polymerase (PARP) inhibitors (PARPi) in WE-68 and SK-ES-1 cells. PARP is another major participant in the DNA damage response [42], making it an important target in the treatment of cancers [43, 44], including paediatric solid malignancies [45, 46], particularly ES [47]. We examined the combination of adavosertib and PARPi in exactly the same way as the combination of adavosertib and triapine, just by substituting a PARPi for triapine. Fig. S1 shows that the two PARPi tested, olaparib and veliparib, elicited cell death and Δ*ψ*_m_ loss in a concentration-dependent manner. These effects were enhanced by the addition of adavosertib. The CI analysis revealed that the majority of adavosertib-PARPi combinations were synergistic, albeit less so than the RNRi-WEE1i combinations (Tables S7 to S10).

### Combination treatment of triapine with WEE1i exacerbates the replication stress response and activates p53

**Fig. 5:**
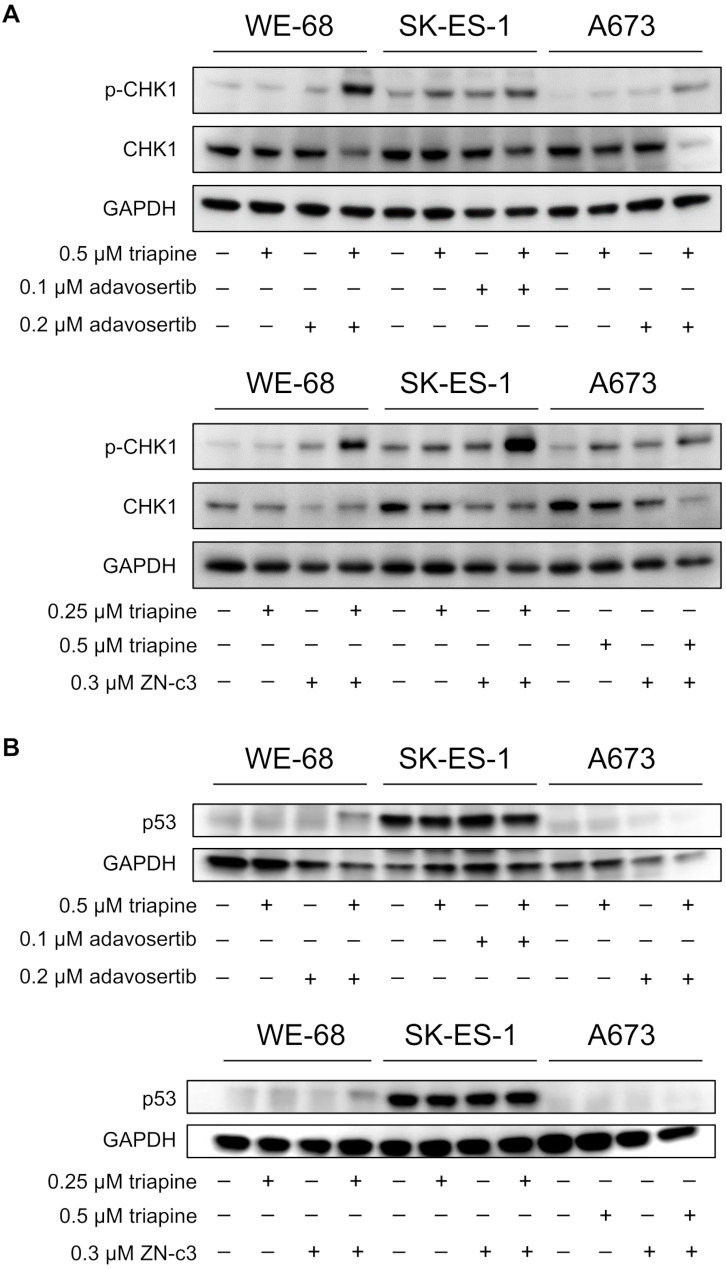
RNRi and WEE1i cooperate in increasing CHK1 phosphorylation and p53 abundance. Cells were exposed to triapine in combination with adavosertib or ZN-c3 for 24 h. (**A**, **B**) p-CHK1, CHK1, p53 and GAPDH abundance were determined by immunoblotting; the blots are representative of each three independent experiments

To gain further insight into the triapine-WEE1i combinations’ mode of action, we asked whether the treatment would interact in enhancing replication stress. Since the phosphorylation of CHK1 is considered a reliable marker of active replication stress [48], we determined phospho-Ser345-CHK1 by immunoblotting. As depicted in Fig. 5A, both triapine-adavosertib and triapine-ZN-c3 boosted CHK1 phosphorylation relative to the single treatments in the three cell lines, thus evidencing augmented replication stress after combination treatment. Furthermore, the combination treatments resulted in a decrease of CHK1 protein expression, most notably in A673 cells.

Given the pivotal role of the tumour suppressor protein p53 in human cancer biology, including response to therapy [49], we also asked whether triapine in combination with WEE1i had an effect on p53 in ES cells. To this end, we determined p53 abundance by immunoblotting and p53 target gene expression by real-time RT-PCR after a 24-h treatment. Figure 5B shows that both triapine-WEE1i combinations increased p53 levels in p53 wild-type WE-68 cells. Missense mutant p53 in SK-ES-1 cells was found to be accumulated to the high level typical of p53 mutant cancers [50], but was not further enhanced by the treatment. p53-deficient A673 cells predictably did not display p53 expression. The combination treatments had no effect on *TP53* gene expression, demonstrating that the effect on p53 abundance occurred at the posttranscriptional level (Fig. S2).

**Fig. 6:**
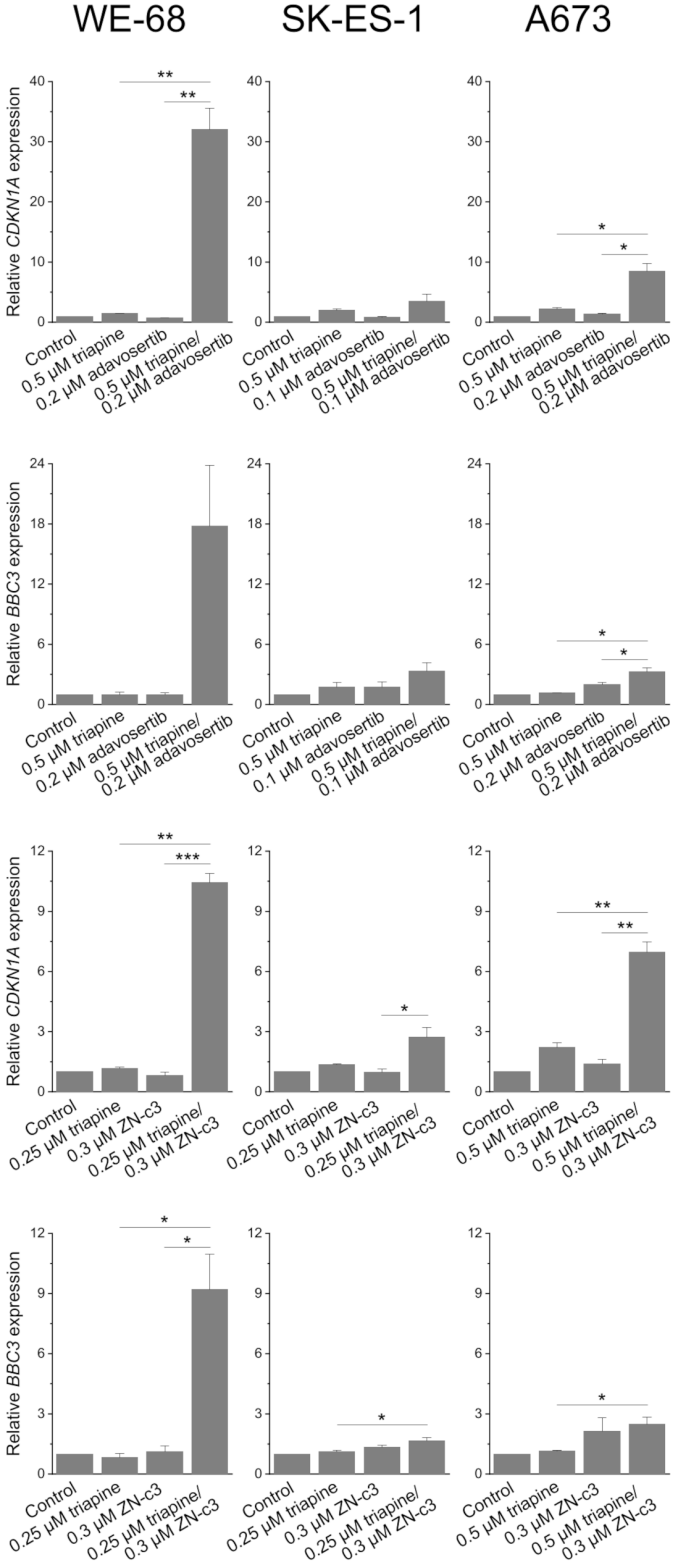
RNRi and WEE1i cooperate in inducing p53 target gene expression. Cells were exposed to triapine in combination with adavosertib or ZN-c3 for 24 h. *CDKN1A* and *BBC3* expression levels were determined by real-time RT-PCR and normalised to *B2M* expression levels; relative gene expression levels are the ratio of treated cells to untreated cells. Means ± SEM of each three independent measurements are shown (*p < 0.05, **p < 0.01, ***p < 0.001)

Figure 6 shows that the increase in p53 abundance was accompanied by a strong induction of two major p53-transactivated genes, *CDKN1A* (encoding the cell cycle-inhibitory protein p21) and *BBC3* (encoding the proapoptotic protein PUMA) [49], in the p53 wild-type cells. Interestingly, triapine-WEE1i combination treatment also led to the induction of *CDKN1A* and *BBC3* expression in the p53 mutant cells, although to a much lower extent than in the p53 wild-type cells.

## Discussion

In this study, we continued our investigation of the combined inhibition of RNR and the ATR/CHK1/WEE1 pathway as a viable option for the treatment of ES. Our previous study on this subject showed that the combination of RNRi with ATRi exerted synergistic anticancer activity in ES [25]. Our present study demonstrates that the combination of RNRi with WEE1i was also synergistically effective against ES. This work thus complements the previous one and further supports the concept of combined targeting of RNR and the ATR pathway as a promising treatment approach for ES. Additional support for the utility of RNRi combined with ATR pathway inhibitors in ES comes from studies on the cooperative action of RNRi with inhibitors of CHK1 [51, 52].

Most notably, we found here that the combination of the RNRi triapine with either WEE1i adavosertib or ZN-c3 greatly enhanced their individual effects. The CI analyses evidenced that the combination effect was indeed synergistic at most drug concentrations tested. Adavosertib has already been shown to be effective in ES [53–57], yet our study is the first to demonstrate the effectiveness of ZN-c3 in ES cells, pointing to a class effect of WEE1i in ES. ZN-c3 offers the potential advantage of better kinase selectivity compared to other WEE1 inhibitors including adavosertib [58].

Although it is known that the inhibition of WEE1 results in RRM2 depletion [59, 60], the mechanism that accounts for the cooperative action of combined ATR/CHK1/WEE1 pathway and RNR inhibition has not yet been conclusively clarified. A plausible explanation, however, is the following: Cancer cells generally suffer from high replication stress [18]. The inhibition of RNR decreases the concentration of dNTPs, further increasing replication stress. This results in the activation of the ATR pathway, which serves to cope with replication stress. ATR pathway inhibitors disable this protective reaction, ultimately causing cancer cell death [61]. Consistent with this explanation, our experiments showed that triapine-WEE1i combination treatment resulted in exacerbated replication stress, as evidenced by greatly enhanced CHK1 phosphorylation after combination treatment. Additional evidence for increased replication stress comes from the finding that the combination treatments reduced CHK1 protein levels, consistent with the observation that replication stress-triggered CHK1 phosphorylation at Ser345 induced the polyubiquitination and degradation of CHK1 [62].

Our experiments further revealed that the cooperative action of triapine-WEE1i combination treatment involved the mitochondrial pathway of apoptosis, as assessed by determining Δ*ψ*_m_ dissipation, caspase 3/7 activation and DNA fragmentation. The different measurements followed a similar pattern, with the exception of caspase 3/7 activation in A673 cells, thus confirming the robustness of the results. The use of the pan-caspase inhibitor z-VAD-fmk further corroborated the induction of apoptosis by triapine-WEE1i, as it reduced cell death and DNA fragmentation. It should be noted, however, that z-VAD-fmk did not fully prevent cell death, implying that the combination treatments harnessed both caspase-dependent and independent cell death pathways. In WE-68 cells, z-VAD-fmk also affected triapine-WEE1i-induced Δ*ψ*_m_ dissipation, suggesting that the mitochondrial apoptotic function depended in part on caspases, possibly as a result of a feedback amplification loop [63]. These results are in agreement with our previous study on the effect of combining triapine with ATRi [25] as well as another study that reported apoptosis induction in response to concomitant ATR pathway and RNR inhibition [53] in ES cells.

In addition, we found the combination effect to be independent of the cells’ p53 mutational status as triapine combined with WEE1i was similarly effective in p53 wild-type, p53 missense mutant and p53-deficient ES cells. Previous studies yielded contradictory results on the impact of p53 status on the sensitivity to WEE1i. Some demonstrated effectiveness of WEE1i selectively in p53 mutant cells [64–66], whereas others reported no association between p53 functionality and responsiveness to WEE1i [22, 67, 68]. These inconsistencies were explained by differences in the intrinsic chromosomal instability of the tumours examined [32]. In any case, the p53-independent action of RNRi-WEE1i combination treatment in ES cells is an important result from the clinical perspective. Mutations in *TP53* are rare in ES, but the ∼ 7% of ES patients with mutant *TP53* are relatively insensitive to chemotherapy and radiotherapy and have a worse than average outcome [69–73]. Somatic mutations are generally infrequent in ES, with *STAG2* being the most commonly mutated gene (∼ 17% of cases) [70–72]. (STAG2 is a subunit of the cohesin complex, and its inactivation can cause aneuploidy in cancer [74]). Co-occurrence of *TP53* and *STAG2* mutations is associated with a dismal prognosis in ES [72], patients with these mutations are therefore particularly in need for new therapies. We observed RNRi-WEE1i combination treatment to be effective in *TP53*/*STAG2* double-mutant SK-ES-1 cells, indicating that it may be an option also for these difficult-to-treat cases.

Yet we also noted that triapine-WEE1i combination treatment produced an increase in p53 abundance and a strongly enhanced expression of the p53 target genes *CDKN1A* and *BBC3* in p53 wild-type ES cells. The activation of the p53 pathway may thus contribute to the cytotoxic effect of combined RNR and WEE1 inhibition in p53-proficient cells. Noteworthy, triapine-WEE1i-induced *CDKN1A* and *BBC3* expression was not restricted to p53 wild-type cells, but also occurred, albeit to a lesser degree, in mutant p53 ES cells. This result implies that not only did triapine-WEE1i kill ES cells independently of functional p53, but it also provoked gene expression in a p53-independent manner, just like the combination of triapine with ATRi [25].

We also assessed the combination of WEE1i with PARPi, with the following rationale: ‘BRCAness’ tumours including ES are considered to be particularly susceptible to PARPi [75], and olaparib was found to be highly effective against ES in vitro [76–78]. However, no objective clinical response was observed in a phase II trial of olaparib in ES patients [79], suggesting that PARPi need to be combined with other agents to achieve a clinical response in ES [47]. Since PARPi cause replication stress and activate the ATR pathway [18], the targeting of the latter by inhibiting ATR, CHK1 or WEE1 is a rational approach to overcome PARPi resistance [80, 81]. This approach, however, has not yet been tested in ES. Our measurements showed that the combination of WEE1i with PARPi was also synergistically active in ES cells, but less so than the combination of WEE1i with RNRi. These data therefore warrant a more in-depth assessment of WEE1i-PARPi combination treatment in ES.

This study has some limitations. Its aim was to investigate whether the combination of RNRi with WEE1i could exert synergistic antineoplastic effects on ES cells. It was focused on drugs that had already been tested in clinical trials to facilitate clinical translation of the findings. Therefore, our study did not attempt to dissect the on-target mechanism of combined RNR-WEE1 inhibition. Additional investigations, such as RRM2 or WEE1 knockdown, RNA interference or CRISPR-based gene editing, will be required to clarify this point. Furthermore, since our study was restricted to in vitro investigations, we cannot make any statement about potential adverse side effects of combined RNRi-WEE1i treatment. Future xenograft studies may shed light on the toxicity of this drug combination.

## Conclusion

This study suggests that the combination of RNRi and WEE1i may be an effective strategy for the therapy of ES. It thus provides a basis for preclinical in vivo and potentially clinical development of this drug combination. Since the combination of RNRi with either ATRi [25] or WEE1i (this study) showed similar anti-ES activity in vitro, a relevant clinical question will be which combination might be more effective and/or less systemically toxic.

## Electronic supplementary material

Below is the link to the electronic supplementary material.

**Additional file S1**: **Table S1**: CI values for triapine plus adavosertib in WE-68 cells. Based on data from Fig. 1A, CI values were calculated with the Chou-Talalay method. CI values in bold indicate a synergistic interaction. **Additional file S2**: **Table S2**: CI values for triapine plus adavosertib in SK-ES-1 cells. Based on data from Fig. 1A, CI values were calculated with the Chou-Talalay method. CI values in bold indicate a synergistic interaction (CI values > 0.8 were not considered synergistic). **Additional file S3**: **Table S3**: CI values for triapine plus adavosertib in A673 cells. Based on data from Fig. 1A, CI values were calculated with the Chou-Talalay method. CI values in bold indicate a synergistic interaction (CI values > 0.8 were not considered synergistic). **Additional file S4**: **Table S4**: CI values for triapine plus ZN-c3 in WE-68 cells. Based on data from Fig. 1B, CI values were calculated with the Chou-Talalay method. CI values in bold indicate a synergistic interaction (CI values > 0.8 were not considered synergistic). **Additional file S5**: **Table S5**: CI values for triapine plus ZN-c3 in SK-ES-1 cells. Based on data from Fig. 1B, CI values were calculated with the Chou-Talalay method. CI values in bold indicate a synergistic interaction (CI values > 0.8 were not considered synergistic). **Additional file S6**: **Table S6**: CI values for triapine plus ZN-c3 in A673 cells. Based on data from Fig. 1B, CI values were calculated with the Chou-Talalay method. CI values in bold indicate a synergistic interaction (CI values > 0.8 were not considered synergistic). **Additional file S9**: **Table S7**: CI values for olaparib plus adavosertib in WE-68 cells. Based on data from Fig. S1, CI values were calculated with the Chou-Talalay method. CI values in bold indicate a synergistic interaction (CI values > 0.8 were not considered synergistic). **Additional file S10**: **Table S8**: CI values for veliparib plus adavosertib in WE-68 cells. Based on data from Fig. S1, CI values were calculated with the Chou-Talalay method. CI values in bold indicate a synergistic interaction (CI values > 0.8 were not considered synergistic). **Additional file S11**: **Table S9**: CI values for olaparib plus adavosertib in SK-ES-1 cells. Based on data from Fig. S1, CI values were calculated with the Chou-Talalay method. CI values in bold indicate a synergistic interaction (CI values > 0.8 were not considered synergistic). **Additional file S12**: **Table S10**: CI values for veliparib plus adavosertib in SK-ES-1 cells. Based on data from Fig. S1, CI values were calculated with the Chou-Talalay method. CI values in bold indicate a synergistic interaction (CI values > 0.8 were not considered synergistic). **Additional file S13**: **Table S11**: Statistical analysis for triapine-adavosertib-induced cell death. Based on data from Fig. 1A, *p* values were calculated with the Kruskal-Wallis test followed by the Dunn’s test. **Additional file S14**: **Table S12**: Statistical analysis for triapine-ZN-c3-induced cell death. Based on data from Fig. 1B, *p* values were calculated with the Kruskal-Wallis test followed by the Dunn’s test. **Additional file S15**: **Table S13**: Statistical analysis for triapine-adavosertib-induced Δ*ψ*_m_ loss. Based on data from Fig. 3A, *p* values were calculated with the Kruskal-Wallis test followed by the Dunn’s test. **Additional file S16**: **Table S14**: Statistical analysis for triapine-ZN-c3-induced Δ*ψ*_m_ loss. Based on data from Fig. 4A, *p* values were calculated with the Kruskal-Wallis test followed by the Dunn’s test. **Additional file S17**: **Table S15**: Statistical analysis for triapine-adavosertib-induced caspase 3/7 activity. Based on data from Fig. 3B, *p* values were calculated with the Kruskal-Wallis test followed by the Dunn’s test. **Additional file S18**: **Table S16**: Statistical analysis for triapine-ZN-c3-induced caspase 3/7 activity. Based on data from Fig. 4B, *p* values were calculated with the Kruskal-Wallis test followed by the Dunn’s test

**Additional file S7**: **Figure S1**: Effects of PARP inhibitors and adavosertib in Ewing’s sarcoma cells. Cells were exposed to drugs for 48 h. (**A**) Cell death and (**B**) Δ*ψ*_m_ loss were determined by flow-cytometric analysis of propidium iodide uptake and DiOC_6_(3) staining, respectively. Means ± SEM of each three independent measurements are shown. **Additional file S8**: **Figure S2**: RNRi and WEE1i do not affect TP53 expression. Cells were exposed to triapine in combination with adavosertib or ZN-c3 for 24 h. *TP53* expression levels were determined by real-time RT-PCR and normalised to *B2M* expression levels; relative gene expression levels are the ratio of treated cells to untreated cells. Means ± SEM of each three independent measurements are shown

Supplementary Material 3

## Data Availability

All data generated or analysed during this study are included in this published article and its supplementary information file. The datasets used and/or analysed during the current study are available from the corresponding author on reasonable request.

## Abbreviations

Ac: DEVD-AMC Acetyl-Asp-Glu-Val-Asp-amido-4-methyl-coumarin
ATRi: ATR inhibitor
CI: Combination index
CST: Cell Signaling Technology
DiOC_6_(3): 3,3’-dihexyloxacarbocyanine iodide
dNTPs: Deoxyribonucleotides
ES: Ewing’s sarcoma
PARP: Poly(ADP-ribose)-polymerase
PARPi: Poly(ADP-ribose)-polymerase inhibitor
PI: Propidium iodide
RNR: Ribonucleotide reductase
RNRi: Ribonucleotide reductase inhibitor
RSR: Replication stress response
