# Supplementary material for: Combined inhibition of ribonucleotide reductase and WEE1 induces synergistic anticancer activity in Ewing’s sarcoma cells
Source: BMC Cancer. 2025 Feb 17;25:277. doi: 10.1186/s12885-025-13691-2 (PMC11831844; doi:10.1186/s12885-025-13691-2)
Supplement: Supplementary file 1 — Additional file S1: Table S1: CI values for triapine plus adavosertib in WE-68 cells. Based on data from Fig. 1A, CI values were calculated with the Chou-Talalay method. CI values in bold indicate a synergistic interaction. Additional file S2: Table S2: CI values for triapine plus adavosertib in SK-ES-1 cells. Based on data from Fig. 1A, CI values were calculated with the Chou-Talalay method. CI values in bold indicate a synergistic interaction (CI values > 0.8 were not considered synergistic). Additional file S3: Table S3: CI values for triapine plus adavosertib in A673 cells. Based on data from Fig. 1A, CI values were calculated with the Chou-Talalay method. CI values in bold indicate a synergistic interaction (CI values > 0.8 were not considered synergistic). Additional file S4: Table S4: CI values for triapine plus ZN-c3 in WE-68 cells. Based on data from Fig. 1B, CI values were calculated with the Chou-Talalay method. CI values in bold indicate a synergistic interaction (CI values > 0.8 were not considered synergistic). Additional file S5: Table S5: CI values for triapine plus ZN-c3 in SK-ES-1 cells. Based on data from Fig. 1B, CI values were calculated with the Chou-Talalay method. CI values in bold indicate a synergistic interaction (CI values > 0.8 were not considered synergistic). Additional file S6: Table S6: CI values for triapine plus ZN-c3 in A673 cells. Based on data from Fig. 1B, CI values were calculated with the Chou-Talalay method. CI values in bold indicate a synergistic interaction (CI values > 0.8 were not considered synergistic). Additional file S9: Table S7: CI values for olaparib plus adavosertib in WE-68 cells. Based on data from Fig. S1, CI values were calculated with the Chou-Talalay method. CI values in bold indicate a synergistic interaction (CI values > 0.8 were not considered synergistic). Additional file S10: Table S8: CI values for veliparib plus adavosertib in WE-68 cells. Based on data from Fig. S1, CI values were calculated with t [file 12885_2025_13691_MOESM1_ESM.docx]

**Additional File 1**

**Table S1. CI values for triapine plus adavosertib in WE-68 cells**

| Triapine (µM) | Adavosertib (µM) | CI |
| --- | --- | --- |
| 0.125 | 0.1 | 2.154 |
| 0.125 | 0.2 | 2.885 |
| 0.125 | 0.5 | **0.182** |
| 0.25 | 0.1 | 3.664 |
| 0.25 | 0.2 | **0.216** |
| 0.25 | 0.5 | **0.064** |
| 0.5 | 0.1 | **0.119** |
| 0.5 | 0.2 | **0.019** |
| 0.5 | 0. 5 | **0.059** |
| 1.0 | 0.1 | **0.010** |
| 1.0 | 0.2 | **0.018** |
| 1.0 | 0.5 | **0.046** |

Based on data from Fig. 1A, CI values were calculated with the Chou-Talalay method. CI values in bold indicate a synergistic interaction.

**Additional File 2**

**Table S2. CI values for triapine plus adavosertib in SK-ES-1 cells**

| Triapine (µM) | Adavosertib (µM) | CI |
| --- | --- | --- |
| 0.125 | 0.05 | 1.121 |
| 0.125 | 0.1 | 1.124 |
| 0.125 | 0.2 | 0.913 |
| 0.25 | 0.05 | **0.468** |
| 0.25 | 0.1 | **0.347** |
| 0.25 | 0.2 | **0.306** |
| 0.5 | 0.05 | **0.649** |
| 0.5 | 0.1 | **0.449** |
| 0.5 | 0.2 | **0.211** |
| 1.0 | 0.05 | **0.453** |
| 1.0 | 0.1 | **0.315** |
| 1.0 | 0.2 | **0.197** |

Based on data from Fig. 1A, CI values were calculated with the Chou-Talalay method. CI values in bold indicate a synergistic interaction (CI values > 0.8 were not considered synergistic).

**Additional File 3**

**Table S3. CI values for triapine plus adavosertib in A673 cells**

| Triapine (µM) | Adavosertib (µM) | CI |
| --- | --- | --- |
| 0.125 | 0.1 | 1.316 |
| 0.125 | 0.2 | 1.450 |
| 0.125 | 0.5 | 0.814 |
| 0.25 | 0.1 | 1.019 |
| 0.25 | 0.2 | **0.733** |
| 0.25 | 0.5 | **0.362** |
| 0.5 | 0.1 | **0.339** |
| 0.5 | 0.2 | **0.179** |
| 0.5 | 0. 5 | **0.161** |
| 1.0 | 0.1 | **0.080** |
| 1.0 | 0.2 | **0.071** |
| 1.0 | 0.5 | **0.144** |

Based on data from Fig. 1A, CI values were calculated with the Chou-Talalay method. CI values in bold indicate a synergistic interaction (CI values > 0.8 were not considered synergistic).

**Additional File 4**

**Table S4. CI values for triapine plus ZN-c3 in WE-68 cells**

| Triapine (µM) | ZN-c3 (µM) | CI |
| --- | --- | --- |
| 0.125 | 0.3 | 1.099 |
| 0.125 | 0.4 | 0.905 |
| 0.125 | 0.5 | **0.561** |
| 0.25 | 0.3 | **0.380** |
| 0.25 | 0.4 | **0.331** |
| 0.25 | 0.5 | **0.407** |
| 0.5 | 0.3 | **0.227** |
| 0.5 | 0.4 | **0.322** |
| 0.5 | 0.5 | **0.392** |

Based on data from Fig. 1B, CI values were calculated with the Chou-Talalay method. CI values in bold indicate a synergistic interaction (CI values > 0.8 were not considered synergistic).

**Additional File 5**

**Table S5. CI values for triapine plus ZN-c3 in SK-ES-1 cells**

| Triapine (µM) | ZN-c3 (µM) | CI |
| --- | --- | --- |
| 0.125 | 0.3 | 1.149 |
| 0.125 | 0.4 | 0.982 |
| 0.125 | 0.5 | 0.803 |
| 0.25 | 0.3 | **0.717** |
| 0.25 | 0.4 | **0.595** |
| 0.25 | 0.5 | **0.474** |
| 0.5 | 0.3 | **0.357** |
| 0.5 | 0.4 | **0.355** |
| 0.5 | 0.5 | **0.383** |

Based on data from Fig. 1B, CI values were calculated with the Chou-Talalay method. CI values in bold indicate a synergistic interaction (CI values > 0.8 were not considered synergistic).

**Additional File 6**

**Table S6. CI values for triapine plus ZN-c3 in A673 cells**

| Triapine (µM) | ZN-c3 (µM) | CI |
| --- | --- | --- |
| 0.125 | 0.3 | 0.955 |
| 0.125 | 0.4 | 1.014 |
| 0.125 | 0.5 | 0.925 |
| 0.25 | 0.3 | **0.644** |
| 0.25 | 0.4 | **0.685** |
| 0.25 | 0.5 | **0.711** |
| 0.5 | 0.3 | **0.374** |
| 0.5 | 0.4 | **0.457** |
| 0.5 | 0.5 | **0.541** |

Based on data from Fig. 1B, CI values were calculated with the Chou-Talalay method. CI values in bold indicate a synergistic interaction (CI values > 0.8 were not considered synergistic).

**Additional File 9**

**Table S7. CI values for olaparib plus adavosertib in WE-68 cells**

| Olaparib (µM) | Adavosertib (µM) | CI |
| --- | --- | --- |
| 0.1 | 0.1 | 1.481 |
| 0.1 | 0.2 | 0.819 |
| 0.1 | 0.5 | **0.345** |
| 0.2 | 0.1 | 1.276 |
| 0.2 | 0.2 | **0.777** |
| 0.2 | 0.5 | **0.318** |
| 0.5 | 0.1 | **0.696** |
| 0.5 | 0.2 | **0.455** |
| 0.5 | 0.5 | **0.300** |
| 1.0 | 0.1 | **0.466** |
| 1.0 | 0.2 | **0.550** |
| 1.0 | 0.5 | **0.692** |

Based on data from Fig. S1A, CI values were calculated with the Chou-Talalay method. CI values in bold indicate a synergistic interaction (CI values > 0.8 were not considered synergistic).

**Additional File 10**

**Table S8. CI values for veliparib plus adavosertib in WE-68 cells**

| Veliparib (µM) | Adavosertib (µM) | CI |
| --- | --- | --- |
| 2.5 | 0.1 | **0,695** |
| 2.5 | 0.2 | 0.989 |
| 2.5 | 0.5 | **0.386** |
| 5.0 | 0.1 | **0.674** |
| 5.0 | 0.2 | **0.510** |
| 5.0 | 0.5 | **0.321** |
| 10.0 | 0.1 | **0.627** |
| 10.0 | 0.2 | **0.547** |
| 10.0 | 0.5 | **0.564** |
| 20.0 | 0.1 | 0.820 |
| 20.0 | 0.2 | 0.847 |
| 20.0 | 0.5 | **0.700** |

Based on data from Fig. S1A, CI values were calculated with the Chou-Talalay method. CI values in bold indicate a synergistic interaction (CI values > 0.8 were not considered synergistic).

**Additional File 11**

**Table S9. CI values for olaparib plus adavosertib in SK-ES-1 cells**

| Olaparib (µM) | Adavosertib (µM) | CI |
| --- | --- | --- |
| 0.25 | 0.05 | 1.291 |
| 0.25 | 0.1 | 1.090 |
| 0.25 | 0.2 | **0.754** |
| 0.5 | 0.05 | 1.002 |
| 0.5 | 0.1 | **0.764** |
| 0.5 | 0.2 | 0.802 |
| 1.0 | 0.05 | **0.701** |
| 1.0 | 0.1 | 0.927 |
| 1.0 | 0. 2 | **0.618** |
| 2.0 | 0.05 | **0.616** |
| 2.0 | 0.1 | **0.573** |
| 2.0 | 0.2 | **0.431** |

Based on data from Fig. S1A, CI values were calculated with the Chou-Talalay method. CI values in bold indicate a synergistic interaction (CI values > 0.8 were not considered synergistic).

**Additional File 12**

**Table S10. CI values for veliparib plus adavosertib in SK-ES-1 cells**

| Veliparib (µM) | Adavosertib (µM) | CI |
| --- | --- | --- |
| 2.5 | 0.05 | 1.567 |
| 2.5 | 0.1 | 1.101 |
| 2.5 | 0.2 | 1.252 |
| 5.0 | 0.05 | 1.128 |
| 5.0 | 0.1 | 0.808 |
| 5.0 | 0.2 | 1.018 |
| 10.0 | 0.05 | **0.790** |
| 10.0 | 0.1 | 0.972 |
| 10.0 | 0.2 | **0.729** |
| 20.0 | 0.05 | **0.607** |
| 20.0 | 0.1 | **0.703** |
| 20.0 | 0.2 | **0.577** |

Based on data from Fig. S1A, CI values were calculated with the Chou-Talalay method. CI values in bold indicate a synergistic interaction (CI values > 0.8 were not considered synergistic).

**Additional File 13**

**Table S11. Statistical analysis for triapine-adavosertib-induced cell death**

WE-68

# Kruskal-Wallis test

| y | n | statistics | df | *p* |
| --- | --- | --- | --- | --- |
| Value | 60 | 54.14461 | 19 | 0.0000314 |

# Dunn's test

| Group 1 | Group 2 | *p* |
| --- | --- | --- |
| 0.125 µM triapine + 0 µM adavosertib | 0.125 µM triapine + 0.1 µM adavosertib | 0.4683990821 |
| 0 µM triapine + 0.1 µM adavosertib | 0.125 µM triapine + 0.1 µM adavosertib | 0.8515672939 |
| 0.25 µM triapine + 0 µM adavosertib | 0.25 µM triapine + 0.1 µM adavosertib | 0.6823026996 |
| 0 µM triapine + 0.1 µM adavosertib | 0.25 µM triapine + 0.1 µM adavosertib | 0.7344942671 |
| 0.5 µM triapine + 0 µM adavosertib | 0.5 µM triapine + 0.1 µM adavosertib | 0.0716995960 |
| 0 µM triapine + 0.1 µM adavosertib | 0.5 µM triapine + 0.1 µM adavosertib | 0.0899301335 |
| 1 µM triapine + 0 µM adavosertib | 1 µM triapine + 0.1 µM adavosertib | 0.0680900031 |
| 0 µM triapine + 0.1 µM adavosertib | 1 µM triapine + 0.1 µM adavosertib | 0.0024537314 |
| 0.125 µM triapine + 0 µM adavosertib | 0.125 µM triapine + 0.2 µM adavosertib | 0.6483161437 |
| 0 µM triapine + 0.2 µM adavosertib | 0.125 µM triapine + 0.2 µM adavosertib | 0.8976390753 |
| 0.25 µM triapine + 0 µM adavosertib | 0.25 µM triapine + 0.2 µM adavosertib | 0.1570427549 |
| 0 µM triapine + 0.2 µM adavosertib | 0.25 µM triapine + 0.2 µM adavosertib | 0.1255132541 |
| 0.5 µM triapine + 0 µM adavosertib | 0.5 µM triapine + 0.2 µM adavosertib | 0.0050038515 |
| 0 µM triapine + 0.2 µM adavosertib | 0.5 µM triapine + 0.2 µM adavosertib | 0.0059903157 |
| 1 µM triapine + 0 µM adavosertib | 1 µM triapine + 0.2 µM adavosertib | 0.0754644864 |
| 0 µM triapine + 0.2 µM adavosertib | 1 µM triapine + 0.2 µM adavosertib | 0.0024537314 |
| 0.125 µM triapine + 0 µM adavosertib | 0.125 µM triapine + 0.5 µM adavosertib | 0.1712156760 |
| 0 µM triapine + 0.5 µM adavosertib | 0.125 µM triapine + 0.5 µM adavosertib | 0.5125214084 |
| 0.25 µM triapine + 0 µM adavosertib | 0.25 µM triapine + 0.5 µM adavosertib | 0.0323406987 |
| 0 µM triapine + 0.5 µM adavosertib | 0.25 µM triapine + 0.5 µM adavosertib | 0.3375662412 |
| 0.5 µM triapine + 0 µM adavosertib | 0.5 µM triapine + 0.5 µM adavosertib | 0.0159893855 |
| 0 µM triapine + 0.5 µM adavosertib | 0.5 µM triapine + 0.5 µM adavosertib | 0.2925497391 |
| 1 µM triapine + 0 µM adavosertib | 1 µM triapine + 0.5 µM adavosertib | 0.1170861717 |
| 0 µM triapine + 0.5 µM adavosertib | 1 µM triapine + 0.5 µM adavosertib | 0.1284249771 |

Based on data from Fig. 1A, *p* values were calculated with the Kruskal-Wallis test followed by the Dunn's test.

SK-ES-1

# Kruskal-Wallis test

| y | n | statistics | df | *p* |
| --- | --- | --- | --- | --- |
| Value | 60 | 58.31464 | 19 | 0.00000713 |

# Dunn's test

| Group 1 | Group 2 | *p* |
| --- | --- | --- |
| 0.125 µM triapine + 0 µM adavosertib | 0.125 µM triap. + 0.05 µM adavosertib | 0.5052333797 |
| 0 µM triapine + 0.05 µM adavosertib | 0.125 µM triap. + 0.05 µM adavosertib | 0.6484802495 |
| 0.25 µM triapine + 0 µM adavosertib | 0.25 µM triapine + 0.05 µM adavosertib | 0.2568609855 |
| 0 µM triapine + 0.05 µM adavosertib | 0.25 µM triapine + 0.05 µM adavosertib | 0.0857426246 |
| 0.5 µM triapine + 0 µM adavosertib | 0.5 µM triapine + 0.05 µM adavosertib | 0.5747455444 |
| 0 µM triapine + 0.05 µM adavosertib | 0.5 µM triapine + 0.05 µM adavosertib | 0.0407966703 |
| 1 µM triapine + 0 µM adavosertib | 1 µM triapine + 0.05 µM adavosertib | 0.6910511157 |
| 0 µM triapine + 0.05 µM adavosertib | 1 µM triapine + 0.05 µM adavosertib | 0.0021104638 |
| 0.125 µM triapine + 0 µM adavosertib | 0.125 µM triapine + 0.1 µM adavosertib | 0.2821973895 |
| 0 µM triapine + 0.1 µM adavosertib | 0.125 µM triapine + 0.1 µM adavosertib | 0.6484802495 |
| 0.25 µM triapine + 0 µM adavosertib | 0.25 µM triapine + 0.1 µM adavosertib | 0.1376725330 |
| 0 µM triapine + 0.1 µM adavosertib | 0.25 µM triapine + 0.1 µM adavosertib | 0.0969445050 |
| 0.5 µM triapine + 0 µM adavosertib | 0.5 µM triapine + 0.1 µM adavosertib | 0.2241124663 |
| 0 µM triapine + 0.1 µM adavosertib | 0.5 µM triapine + 0.1 µM adavosertib | 0.0219598607 |
| 1 µM triapine + 0 µM adavosertib | 1 µM triapine + 0.1 µM adavosertib | 0.4543988208 |
| 0 µM triapine + 0.1 µM adavosertib | 1 µM triapine + 0.1 µM adavosertib | 0.0025631498 |
| 0.125 µM triapine + 0 µM adavosertib | 0.125 µM triapine + 0.2 µM adavosertib | 0.1092872046 |
| 0 µM triapine + 0.2 µM adavosertib | 0.125 µM triapine + 0.2 µM adavosertib | 0.7790646721 |
| 0.25 µM triapine + 0 µM adavosertib | 0.25 µM triapine + 0.2 µM adavosertib | 0.0737065614 |
| 0 µM triapine + 0.2 µM adavosertib | 0.25 µM triapine + 0.2 µM adavosertib | 0.2067971182 |
| 0.5 µM triapine + 0 µM adavosertib | 0.5 µM triapine + 0.2 µM adavosertib | 0.0969445050 |
| 0 µM triapine + 0.2 µM adavosertib | 0.5 µM triapine + 0.2 µM adavosertib | 0.0419615299 |
| 1 µM triapine + 0 µM adavosertib | 1 µM triapine + 0.2 µM adavosertib | 0.3378080230 |
| 0 µM triapine + 0.2 µM adavosertib | 1 µM triapine + 0.2 µM adavosertib | 0.0115749917 |

Based on data from Fig. 1A, *p* values were calculated with the Kruskal-Wallis test followed by the Dunn's test.

A673

# Kruskal-Wallis test

| y | n | statistics | df | *p* |
| --- | --- | --- | --- | --- |
| Value | 60 | 57.30618 | 19 | 0.0000102 |

# Dunn's test

| Group 1 | Group 2 | *p* |
| --- | --- | --- |
| 0.125 µM triapine + 0 µM adavosertib | 0.125 µM triapine + 0.1 µM adavosertib | 0.5202774650 |
| 0 µM triapine + 0.1 µM adavosertib | 0.125 µM triapine + 0.1 µM adavosertib | 0.8976871819 |
| 0.25 µM triapine + 0 µM adavosertib | 0.25 µM triapine + 0.1 µM adavosertib | 0.3091634188 |
| 0 µM triapine + 0.1 µM adavosertib | 0.25 µM triapine + 0.1 µM adavosertib | 0.3261438142 |
| 0.5 µM triapine + 0 µM adavosertib | 0.5 µM triapine + 0.1 µM adavosertib | 0.1376615966 |
| 0 µM triapine + 0.1 µM adavosertib | 0.5 µM triapine + 0.1 µM adavosertib | 0.0582668285 |
| 1 µM triapine + 0 µM adavosertib | 1 µM triapine + 0.1 µM adavosertib | 0.1677866116 |
| 0 µM triapine + 0.1 µM adavosertib | 1 µM triapine + 0.1 µM adavosertib | 0.0058031488 |
| 0.125 µM triapine + 0 µM adavosertib | 0.125 µM triapine + 0.2 µM adavosertib | 0.1865334556 |
| 0 µM triapine + 0.2 µM adavosertib | 0.125 µM triapine + 0.2 µM adavosertib | 0.9533925231 |
| 0.25 µM triapine + 0 µM adavosertib | 0.25 µM triapine + 0.2 µM adavosertib | 0.1256920024 |
| 0 µM triapine + 0.2 µM adavosertib | 0.25 µM triapine + 0.2 µM adavosertib | 0.5278912304 |
| 0.5 µM triapine + 0 µM adavosertib | 0.5 µM triapine + 0.2 µM adavosertib | 0.0364022954 |
| 0 µM triapine + 0.2 µM adavosertib | 0.5 µM triapine + 0.2 µM adavosertib | 0.1017312355 |
| 1 µM triapine + 0 µM adavosertib | 1 µM triapine + 0.2 µM adavosertib | 0.0836243358 |
| 0 µM triapine + 0.2 µM adavosertib | 1 µM triapine + 0.2 µM adavosertib | 0.0248087689 |
| 0.125 µM triapine + 0 µM adavosertib | 0.125 µM triapine + 0.5 µM adavosertib | 0.0104677698 |
| 0 µM triapine + 0.5 µM adavosertib | 0.125 µM triapine + 0.5 µM adavosertib | 0.8516366245 |
| 0.25 µM triapine + 0 µM adavosertib | 0.25 µM triapine + 0.5 µM adavosertib | 0.0091408932 |
| 0 µM triapine + 0.5 µM adavosertib | 0.25 µM triapine + 0.5 µM adavosertib | 0.4758121926 |
| 0.5 µM triapine + 0 µM adavosertib | 0.5 µM triapine + 0.5 µM adavosertib | 0.0069286433 |
| 0 µM triapine + 0.5 µM adavosertib | 0.5 µM triapine + 0.5 µM adavosertib | 0.2110193965 |
| 1 µM triapine + 0 µM adavosertib | 1 µM triapine + 0.5 µM adavosertib | 0.0614413077 |
| 0 µM triapine + 0.5 µM adavosertib | 1 µM triapine + 0.5 µM adavosertib | 0.1642132236 |

Based on data from Fig. 1A, *p* values were calculated with the Kruskal-Wallis test followed by the Dunn's test.

**Additional File 14**

**Table S12. Statistical analysis for triapine-ZN-c3-induced cell death**

WE-68

# Kruskal-Wallis test

| y | n | statistics | df | *p* |
| --- | --- | --- | --- | --- |
| Value | 51 | 44.83832 | 15 | 0.0000812 |

# Dunn's test

| Group 1 | Group 2 | *p* |
| --- | --- | --- |
| 0.125 µM triapine + 0 µM ZN-c3 | 0.125 µM triapine + 0.3 µM ZN-c3 | 0.5996492998 |
| 0 µM triapine + 0.3 µM ZN-c3 | 0.125 µM triapine + 0.3 µM ZN-c3 | 0.7705818956 |
| 0.25 µM triapine + 0 µM ZN-c3 | 0.25 µM triapine + 0.3 µM ZN-c3 | 0.0309323116 |
| 0 µM triapine + 0.3 µM ZN-c3 | 0.25 µM triapine + 0.3 µM ZN-c3 | 0.2678045640 |
| 0.5 µM triapine + 0 µM ZN-c3 | 0.5 µM triapine + 0.3 µM ZN-c3 | 0.0037131243 |
| 0 µM triapine + 0.3 µM ZN-c3 | 0.5 µM triapine + 0.3 µM ZN-c3 | 0.0174705257 |
| 0.125 µM triapine + 0 µM ZN-c3 | 0.125 µM triapine + 0.4 µM ZN-c3 | 0.1705043527 |
| 0 µM triapine + 0.4 µM ZN-c3 | 0.125 µM triapine + 0.4 µM ZN-c3 | 0.6830832648 |
| 0.25 µM triapine + 0 µM ZN-c3 | 0.25 µM triapine + 0.4 µM ZN-c3 | 0.0029350364 |
| 0 µM triapine + 0.4 µM ZN-c3 | 0.25 µM triapine + 0.4 µM ZN-c3 | 0.0752652759 |
| 0.5 µM triapine + 0 µM ZN-c3 | 0.5 µM triapine + 0.4 µM ZN-c3 | 0.0111794061 |
| 0 µM triapine + 0.4 µM ZN-c3 | 0.5 µM triapine + 0.4 µM ZN-c3 | 0.0619954129 |
| 0.125 µM triapine + 0 µM ZN-c3 | 0.125 µM triapine + 0.5 µM ZN-c3 | 0.0384094533 |
| 0 µM triapine + 0.5 µM ZN-c3 | 0.125 µM triapine + 0.5 µM ZN-c3 | 0.6200753773 |
| 0.25 µM triapine + 0 µM ZN-c3 | 0.25 µM triapine + 0.5 µM ZN-c3 | 0.0024230906 |
| 0 µM triapine + 0.5 µM ZN-c3 | 0.25 µM triapine + 0.5 µM ZN-c3 | 0.2206588346 |
| 0.5 µM triapine + 0 µM ZN-c3 | 0.5 µM triapine + 0.5 µM ZN-c3 | 0.0063992622 |
| 0 µM triapine + 0.5 µM ZN-c3 | 0.5 µM triapine + 0.5 µM ZN-c3 | 0.1488829133 |

Based on data from Fig. 1B, *p* values were calculated with the Kruskal-Wallis test followed by the Dunn's test.

SK-ES-1

# Kruskal-Wallis test

| y | n | statistics | df | *p* |
| --- | --- | --- | --- | --- |
| Value | 51 | 46.04757 | 15 | 0.0000523 |

# Dunn's test

| Group 1 | Group 2 | *p* |
| --- | --- | --- |
| 0.125 µM triapine + 0 µM ZN-c3 | 0.125 µM triapine + 0.3 µM ZN-c3 | 0.3737762187 |
| 0 µM triapine + 0.3 µM ZN-c3 | 0.125 µM triapine + 0.3 µM ZN-c3 | 0.9187052068 |
| 0.25 µM triapine + 0 µM ZN-c3 | 0.25 µM triapine + 0.3 µM ZN-c3 | 0.0964729077 |
| 0 µM triapine + 0.3 µM ZN-c3 | 0.25 µM triapine + 0.3 µM ZN-c3 | 0.2434303937 |
| 0.5 µM triapine + 0 µM ZN-c3 | 0.5 µM triapine + 0.3 µM ZN-c3 | 0.0344981624 |
| 0 µM triapine + 0.3 µM ZN-c3 | 0.5 µM triapine + 0.3 µM ZN-c3 | 0.0287349496 |
| 0.125 µM triapine + 0 µM ZN-c3 | 0.125 µM triapine + 0.4 µM ZN-c3 | 0.0907680197 |
| 0 µM triapine + 0.4 µM ZN-c3 | 0.125 µM triapine + 0.4 µM ZN-c3 | 0.6618062357 |
| 0.25 µM triapine + 0 µM ZN-c3 | 0.25 µM triapine + 0.4 µM ZN-c3 | 0.0196528334 |
| 0 µM triapine + 0.4 µM ZN-c3 | 0.25 µM triapine + 0.4 µM ZN-c3 | 0.1705043527 |
| 0.5 µM triapine + 0 µM ZN-c3 | 0.5 µM triapine + 0.4 µM ZN-c3 | 0.0090557090 |
| 0 µM triapine + 0.4 µM ZN-c3 | 0.5 µM triapine + 0.4 µM ZN-c3 | 0.0266733481 |
| 0.125 µM triapine + 0 µM ZN-c3 | 0.125 µM triapine + 0.5 µM ZN-c3 | 0.0167918718 |
| 0 µM triapine + 0.5 µM ZN-c3 | 0.125 µM triapine + 0.5 µM ZN-c3 | 0.6830832648 |
| 0.25 µM triapine + 0 µM ZN-c3 | 0.25 µM triapine + 0.5 µM ZN-c3 | 0.0046744175 |
| 0 µM triapine + 0.5 µM ZN-c3 | 0.25 µM triapine + 0.5 µM ZN-c3 | 0.2554154190 |
| 0.5 µM triapine + 0 µM ZN-c3 | 0.5 µM triapine + 0.5 µM ZN-c3 | 0.0048918040 |
| 0 µM triapine + 0.5 µM ZN-c3 | 0.5 µM triapine + 0.5 µM ZN-c3 | 0.0907680197 |

Based on data from Fig. 1B, *p* values were calculated with the Kruskal-Wallis test followed by the Dunn's test.

A673

# Kruskal-Wallis test

| y | n | statistics | df | *p* |
| --- | --- | --- | --- | --- |
| Value | 51 | 44.62972 | 15 | 0.0000876 |

# Dunn's test

| Group 1 | Group 2 | *p* |
| --- | --- | --- |
| 0.125 µM triapine + 0 µM ZN-c3 | 0.125 µM triapine + 0.3 µM ZN-c3 | 0.2805744595 |
| 0 µM triapine + 0.3 µM ZN-c3 | 0.125 µM triapine + 0.3 µM ZN-c3 | 0.9418797922 |
| 0.25 µM triapine + 0 µM ZN-c3 | 0.25 µM triapine + 0.3 µM ZN-c3 | 0.0801566291 |
| 0 µM triapine + 0.3 µM ZN-c3 | 0.25 µM triapine + 0.3 µM ZN-c3 | 0.3073947791 |
| 0.5 µM triapine + 0 µM ZN-c3 | 0.5 µM triapine + 0.3 µM ZN-c3 | 0.0086733749 |
| 0 µM triapine + 0.3 µM ZN-c3 | 0.5 µM triapine + 0.3 µM ZN-c3 | 0.0266651146 |
| 0.125 µM triapine + 0 µM ZN-c3 | 0.125 µM triapine + 0.4 µM ZN-c3 | 0.1153025712 |
| 0 µM triapine + 0.4 µM ZN-c3 | 0.125 µM triapine + 0.4 µM ZN-c3 | 0.9187008091 |
| 0.25 µM triapine + 0 µM ZN-c3 | 0.25 µM triapine + 0.4 µM ZN-c3 | 0.0196461863 |
| 0 µM triapine + 0.4 µM ZN-c3 | 0.25 µM triapine + 0.4 µM ZN-c3 | 0.2553896198 |
| 0.5 µM triapine + 0 µM ZN-c3 | 0.5 µM triapine + 0.4 µM ZN-c3 | 0.0046721755 |
| 0 µM triapine + 0.4 µM ZN-c3 | 0.5 µM triapine + 0.4 µM ZN-c3 | 0.0507108254 |
| 0.125 µM triapine + 0 µM ZN-c3 | 0.125 µM triapine + 0.5 µM ZN-c3 | 0.0161307043 |
| 0 µM triapine + 0.5 µM ZN-c3 | 0.125 µM triapine + 0.5 µM ZN-c3 | 0.8610970778 |
| 0.25 µM triapine + 0 µM ZN-c3 | 0.25 µM triapine + 0.5 µM ZN-c3 | 0.0061193091 |
| 0 µM triapine + 0.5 µM ZN-c3 | 0.25 µM triapine + 0.5 µM ZN-c3 | 0.4310476894 |
| 0.5 µM triapine + 0 µM ZN-c3 | 0.5 µM triapine + 0.5 µM ZN-c3 | 0.0029334921 |
| 0 µM triapine + 0.5 µM ZN-c3 | 0.5 µM triapine + 0.5 µM ZN-c3 | 0.1797593173 |

Based on data from Fig. 1B, *p* values were calculated with the Kruskal-Wallis test followed by the Dunn's test.

**Additional File 15**

**Table S13. Statistical analysis for triapine-adavosertib-induced Δ*ψ*_m_ loss**

WE-68

# Kruskal-Wallis test

| y | n | statistics | df | *p* |
| --- | --- | --- | --- | --- |
| Value | 60 | 54.63175 | 19 | 0.0000265 |

# Dunn's test

| Group 1 | Group 2 | *p* |
| --- | --- | --- |
| 0.125 µM triapine + 0 µM adavosertib | 0.125 µM triapine + 0.1 µM adavosertib | 0.5747561730 |
| 0 µM triapine + 0.1 µM adavosertib | 0.125 µM triapine + 0.1 µM adavosertib | 0.8333572452 |
| 0.25 µM triapine + 0 µM adavosertib | 0.25 µM triapine + 0.1 µM adavosertib | 0.8151594396 |
| 0 µM triapine + 0.1 µM adavosertib | 0.25 µM triapine + 0.1 µM adavosertib | 0.7170892372 |
| 0.5 µM triapine + 0 µM adavosertib | 0.5 µM triapine + 0.1 µM adavosertib | 0.3036623924 |
| 0 µM triapine + 0.1 µM adavosertib | 0.5 µM triapine + 0.1 µM adavosertib | 0.0614557359 |
| 1 µM triapine + 0 µM adavosertib | 1 µM triapine + 0.1 µM adavosertib | 0.4000169190 |
| 0 µM triapine + 0.1 µM adavosertib | 1 µM triapine + 0.1 µM adavosertib | 0.0206471447 |
| 0.125 µM triapine + 0 µM adavosertib | 0.125 µM triapine + 0.2 µM adavosertib | 0.6910592585 |
| 0 µM triapine + 0.2 µM adavosertib | 0.125 µM triapine + 0.2 µM adavosertib | 0.9533951106 |
| 0.25 µM triapine + 0 µM adavosertib | 0.25 µM triapine + 0.2 µM adavosertib | 0.1439892306 |
| 0 µM triapine + 0.2 µM adavosertib | 0.25 µM triapine + 0.2 µM adavosertib | 0.0900999331 |
| 0.5 µM triapine + 0 µM adavosertib | 0.5 µM triapine + 0.2 µM adavosertib | 0.0923398575 |
| 0 µM triapine + 0.2 µM adavosertib | 0.5 µM triapine + 0.2 µM adavosertib | 0.0085391919 |
| 1 µM triapine + 0 µM adavosertib | 1 µM triapine + 0.2 µM adavosertib | 0.0946242868 |
| 0 µM triapine + 0.2 µM adavosertib | 1 µM triapine + 0.2 µM adavosertib | 0.0011561766 |
| 0.125 µM triapine + 0 µM adavosertib | 0.125 µM triapine + 0.5 µM adavosertib | 0.0923398575 |
| 0 µM triapine + 0.5 µM adavosertib | 0.125 µM triapine + 0.5 µM adavosertib | 0.4000169190 |
| 0.25 µM triapine + 0 µM adavosertib | 0.25 µM triapine + 0.5 µM adavosertib | 0.0104720561 |
| 0 µM triapine + 0.5 µM adavosertib | 0.25 µM triapine + 0.5 µM adavosertib | 0.1346114943 |
| 0.5 µM triapine + 0 µM adavosertib | 0.5 µM triapine + 0.5 µM adavosertib | 0.0263600939 |
| 0 µM triapine + 0.5 µM adavosertib | 0.5 µM triapine + 0.5 µM adavosertib | 0.0614557359 |
| 1 µM triapine + 0 µM adavosertib | 1 µM triapine + 0.5 µM adavosertib | 0.1200400223 |
| 0 µM triapine + 0.5 µM adavosertib | 1 µM triapine + 0.5 µM adavosertib | 0.0664855457 |

Based on data from Fig. 2A, *p* values were calculated with the Kruskal-Wallis test followed by the Dunn's test.

SK-ES-1

# Kruskal-Wallis test

| y | n | statistics | df | *p* |
| --- | --- | --- | --- | --- |
| Value | 60 | 57.90707 | 19 | 0.00000825 |

# Dunn's test

| Group 1 | Group 2 | *p* |
| --- | --- | --- |
| 0.125 µM triapine + 0 µM adavosertib | 0.125 µM triap. + 0.05 µM adavosertib | 0.40003656066 |
| 0 µM triapine + 0.05 µM adavosertib | 0.125 µM triap. + 0.05 µM adavosertib | 0.54332649662 |
| 0.25 µM triapine + 0 µM adavosertib | 0.25 µM triapine + 0.05 µM adavosertib | 0.29282481895 |
| 0 µM triapine + 0.05 µM adavosertib | 0.25 µM triapine + 0.05 µM adavosertib | 0.09696770957 |
| 0.5 µM triapine + 0 µM adavosertib | 0.5 µM triapine + 0.05 µM adavosertib | 0.59081128879 |
| 0 µM triapine + 0.05 µM adavosertib | 0.5 µM triapine + 0.05 µM adavosertib | 0.03150439199 |
| 1 µM triapine + 0 µM adavosertib | 1 µM triapine + 0.05 µM adavosertib | 0.67391721994 |
| 0 µM triapine + 0.05 µM adavosertib | 1 µM triapine + 0.05 µM adavosertib | 0.00147676382 |
| 0.125 µM triapine + 0 µM adavosertib | 0.125 µM triapine + 0.1 µM adavosertib | 0.21536129116 |
| 0 µM triapine + 0.1 µM adavosertib | 0.125 µM triapine + 0.1 µM adavosertib | 0.50526295920 |
| 0.25 µM triapine + 0 µM adavosertib | 0.25 µM triapine + 0.1 µM adavosertib | 0.16073817098 |
| 0 µM triapine + 0.1 µM adavosertib | 0.25 µM triapine + 0.1 µM adavosertib | 0.09463803721 |
| 0.5 µM triapine + 0 µM adavosertib | 0.5 µM triapine + 0.1 µM adavosertib | 0.38707584180 |
| 0 µM triapine + 0.1 µM adavosertib | 0.5 µM triapine + 0.1 µM adavosertib | 0.03243933895 |
| 1 µM triapine + 0 µM adavosertib | 1 µM triapine + 0.1 µM adavosertib | 0.52793136627 |
| 0 µM triapine + 0.1 µM adavosertib | 1 µM triapine + 0.1 µM adavosertib | 0.00228353979 |
| 0.125 µM triapine + 0 µM adavosertib | 0.125 µM triapine + 0.2 µM adavosertib | 0.13462774171 |
| 0 µM triapine + 0.2 µM adavosertib | 0.125 µM triapine + 0.2 µM adavosertib | 0.59890734038 |
| 0.25 µM triapine + 0 µM adavosertib | 0.25 µM triapine + 0.2 µM adavosertib | 0.10674837120 |
| 0 µM triapine + 0.2 µM adavosertib | 0.25 µM triapine + 0.2 µM adavosertib | 0.13769987291 |
| 0.5 µM triapine + 0 µM adavosertib | 0.5 µM triapine + 0.2 µM adavosertib | 0.17515004154 |
| 0 µM triapine + 0.2 µM adavosertib | 0.5 µM triapine + 0.2 µM adavosertib | 0.02558451698 |
| 1 µM triapine + 0 µM adavosertib | 1 µM triapine + 0.2 µM adavosertib | 0.40003656066 |
| 0 µM triapine + 0.2 µM adavosertib | 1 µM triapine + 0.2 µM adavosertib | 0.00418819757 |

Based on data from Fig. 2A, *p* values were calculated with the Kruskal-Wallis test followed by the Dunn's test.

A673

# Kruskal-Wallis test

| y | n | statistics | df | *p* |
| --- | --- | --- | --- | --- |
| Value | 60 | 57.33757 | 19 | 0.0000101 |

# Dunn's test

| Group 1 | Group 2 | *p* |
| --- | --- | --- |
| 0.125 µM triapine + 0 µM adavosertib | 0.125 µM triapine + 0.1 µM adavosertib | 0.5127499921 |
| 0 µM triapine + 0.1 µM adavosertib | 0.125 µM triapine + 0.1 µM adavosertib | 1.0000000000 |
| 0.25 µM triapine + 0 µM adavosertib | 0.25 µM triapine + 0.1 µM adavosertib | 0.2472022703 |
| 0 µM triapine + 0.1 µM adavosertib | 0.25 µM triapine + 0.1 µM adavosertib | 0.5988971855 |
| 0.5 µM triapine + 0 µM adavosertib | 0.5 µM triapine + 0.1 µM adavosertib | 0.0836458104 |
| 0 µM triapine + 0.1 µM adavosertib | 0.5 µM triapine + 0.1 µM adavosertib | 0.1067386337 |
| 1 µM triapine + 0 µM adavosertib | 1 µM triapine + 0.1 µM adavosertib | 0.1678161446 |
| 0 µM triapine + 0.1 µM adavosertib | 1 µM triapine + 0.1 µM adavosertib | 0.0141038934 |
| 0.125 µM triapine + 0 µM adavosertib | 0.125 µM triapine + 0.2 µM adavosertib | 0.2241317776 |
| 0 µM triapine + 0.2 µM adavosertib | 0.125 µM triapine + 0.2 µM adavosertib | 0.9906740575 |
| 0.25 µM triapine + 0 µM adavosertib | 0.25 µM triapine + 0.2 µM adavosertib | 0.0682395206 |
| 0 µM triapine + 0.2 µM adavosertib | 0.25 µM triapine + 0.2 µM adavosertib | 0.5203064487 |
| 0.5 µM triapine + 0 µM adavosertib | 0.5 µM triapine + 0.2 µM adavosertib | 0.0248187944 |
| 0 µM triapine + 0.2 µM adavosertib | 0.5 µM triapine + 0.2 µM adavosertib | 0.1145745162 |
| 1 µM triapine + 0 µM adavosertib | 1 µM triapine + 0.2 µM adavosertib | 0.0682395206 |
| 0 µM triapine + 0.2 µM adavosertib | 1 µM triapine + 0.2 µM adavosertib | 0.0188032424 |
| 0.125 µM triapine + 0 µM adavosertib | 0.125 µM triapine + 0.5 µM adavosertib | 0.0194017601 |
| 0 µM triapine + 0.5 µM adavosertib | 0.125 µM triapine + 0.5 µM adavosertib | 0.9069503788 |
| 0.25 µM triapine + 0 µM adavosertib | 0.25 µM triapine + 0.5 µM adavosertib | 0.0043442528 |
| 0 µM triapine + 0.5 µM adavosertib | 0.25 µM triapine + 0.5 µM adavosertib | 0.5127499921 |
| 0.5 µM triapine + 0 µM adavosertib | 0.5 µM triapine + 0.5 µM adavosertib | 0.0032240435 |
| 0 µM triapine + 0.5 µM adavosertib | 0.5 µM triapine + 0.5 µM adavosertib | 0.2068160436 |
| 1 µM triapine + 0 µM adavosertib | 1 µM triapine + 0.5 µM adavosertib | 0.0582843033 |
| 0 µM triapine + 0.5 µM adavosertib | 1 µM triapine + 0.5 µM adavosertib | 0.1607265425 |

Based on data from Fig. 2A, *p* values were calculated with the Kruskal-Wallis test followed by the Dunn's test.

**Additional File 16**

**Table S14. Statistical analysis for triapine-ZN-c3-induced Δ*ψ*_m_ loss**

WE-68

# Kruskal-Wallis test

| y | n | statistics | df | *p* |
| --- | --- | --- | --- | --- |
| Value | 51 | 45.10769 | 15 | 0.0000736 |

# Dunn's test

| Group 1 | Group 2 | *p* |
| --- | --- | --- |
| 0.125 µM triapine + 0 µM ZN-c3 | 0.125 µM triapine + 0.3 µM ZN-c3 | 0.4483090361 |
| 0 µM triapine + 0.3 µM ZN-c3 | 0.125 µM triapine + 0.3 µM ZN-c3 | 0.9534890110 |
| 0.25 µM triapine + 0 µM ZN-c3 | 0.25 µM triapine + 0.3 µM ZN-c3 | 0.0309232039 |
| 0 µM triapine + 0.3 µM ZN-c3 | 0.25 µM triapine + 0.3 µM ZN-c3 | 0.1530084404 |
| 0.5 µM triapine + 0 µM ZN-c3 | 0.5 µM triapine + 0.3 µM ZN-c3 | 0.0320747322 |
| 0 µM triapine + 0.3 µM ZN-c3 | 0.5 µM triapine + 0.3 µM ZN-c3 | 0.0220623413 |
| 0.125 µM triapine + 0 µM ZN-c3 | 0.125 µM triapine + 0.4 µM ZN-c3 | 0.1153025712 |
| 0 µM triapine + 0.4 µM ZN-c3 | 0.125 µM triapine + 0.4 µM ZN-c3 | 0.5795128975 |
| 0.25 µM triapine + 0 µM ZN-c3 | 0.25 µM triapine + 0.4 µM ZN-c3 | 0.0063963926 |
| 0 µM triapine + 0.4 µM ZN-c3 | 0.25 µM triapine + 0.4 µM ZN-c3 | 0.0935677924 |
| 0.5 µM triapine + 0 µM ZN-c3 | 0.5 µM triapine + 0.4 µM ZN-c3 | 0.0121411469 |
| 0 µM triapine + 0.4 µM ZN-c3 | 0.5 µM triapine + 0.4 µM ZN-c3 | 0.0196461863 |
| 0.125 µM triapine + 0 µM ZN-c3 | 0.125 µM triapine + 0.5 µM ZN-c3 | 0.0309232039 |
| 0 µM triapine + 0.5 µM ZN-c3 | 0.125 µM triapine + 0.5 µM ZN-c3 | 0.6200563883 |
| 0.25 µM triapine + 0 µM ZN-c3 | 0.25 µM triapine + 0.5 µM ZN-c3 | 0.0009329707 |
| 0 µM triapine + 0.5 µM ZN-c3 | 0.25 µM triapine + 0.5 µM ZN-c3 | 0.1055452734 |
| 0.5 µM triapine + 0 µM ZN-c3 | 0.5 µM triapine + 0.5 µM ZN-c3 | 0.0320747322 |
| 0 µM triapine + 0.5 µM ZN-c3 | 0.5 µM triapine + 0.5 µM ZN-c3 | 0.1845368085 |

Based on data from Fig. 3A, *p* values were calculated with the Kruskal-Wallis test followed by the Dunn's test.

SK-ES-1

# Kruskal-Wallis test

| y | n | statistics | df | *p* |
| --- | --- | --- | --- | --- |
| Value | 51 | 46.64626 | 15 | 0.0000419 |

# Dunn's test

| Group 1 | Group 2 | *p* |
| --- | --- | --- |
| 0.125 µM triapine + 0 µM ZN-c3 | 0.125 µM triapine + 0.3 µM ZN-c3 | 0.44834598805 |
| 0 µM triapine + 0.3 µM ZN-c3 | 0.125 µM triapine + 0.3 µM ZN-c3 | 0.88407693844 |
| 0.25 µM triapine + 0 µM ZN-c3 | 0.25 µM triapine + 0.3 µM ZN-c3 | 0.07527319310 |
| 0 µM triapine + 0.3 µM ZN-c3 | 0.25 µM triapine + 0.3 µM ZN-c3 | 0.14483238193 |
| 0.5 µM triapine + 0 µM ZN-c3 | 0.5 µM triapine + 0.3 µM ZN-c3 | 0.09077678148 |
| 0 µM triapine + 0.3 µM ZN-c3 | 0.5 µM triapine + 0.3 µM ZN-c3 | 0.01550646408 |
| 0.125 µM triapine + 0 µM ZN-c3 | 0.125 µM triapine + 0.4 µM ZN-c3 | 0.10875096333 |
| 0 µM triapine + 0.4 µM ZN-c3 | 0.125 µM triapine + 0.4 µM ZN-c3 | 0.59965920321 |
| 0.25 µM triapine + 0 µM ZN-c3 | 0.25 µM triapine + 0.4 µM ZN-c3 | 0.01817625162 |
| 0 µM triapine + 0.4 µM ZN-c3 | 0.25 µM triapine + 0.4 µM ZN-c3 | 0.11533217927 |
| 0.5 µM triapine + 0 µM ZN-c3 | 0.5 µM triapine + 0.4 µM ZN-c3 | 0.03576733242 |
| 0 µM triapine + 0.4 µM ZN-c3 | 0.5 µM triapine + 0.4 µM ZN-c3 | 0.01817625162 |
| 0.125 µM triapine + 0 µM ZN-c3 | 0.125 µM triapine + 0.5 µM ZN-c3 | 0.01679484032 |
| 0 µM triapine + 0.5 µM ZN-c3 | 0.125 µM triapine + 0.5 µM ZN-c3 | 0.64080793065 |
| 0.25 µM triapine + 0 µM ZN-c3 | 0.25 µM triapine + 0.5 µM ZN-c3 | 0.00560122055 |
| 0 µM triapine + 0.5 µM ZN-c3 | 0.25 µM triapine + 0.5 µM ZN-c3 | 0.25542831768 |
| 0.5 µM triapine + 0 µM ZN-c3 | 0.5 µM triapine + 0.5 µM ZN-c3 | 0.01817625162 |
| 0 µM triapine + 0.5 µM ZN-c3 | 0.5 µM triapine + 0.5 µM ZN-c3 | 0.07527319310 |

Based on data from Fig. 3A, *p* values were calculated with the Kruskal-Wallis test followed by the Dunn's test.

A673

# Kruskal-Wallis test

| y | n | statistics | df | *p* |
| --- | --- | --- | --- | --- |
| Value | 51 | 43.92212 | 15 | 0.000113 |

# Dunn's test

| Group 1 | Group 2 | *p* |
| --- | --- | --- |
| 0.125 µM triapine + 0 µM ZN-c3 | 0.125 µM triapine + 0.3 µM ZN-c3 | 0.1943725640 |
| 0 µM triapine + 0.3 µM ZN-c3 | 0.125 µM triapine + 0.3 µM ZN-c3 | 0.8382434983 |
| 0.25 µM triapine + 0 µM ZN-c3 | 0.25 µM triapine + 0.3 µM ZN-c3 | 0.0752494416 |
| 0 µM triapine + 0.3 µM ZN-c3 | 0.25 µM triapine + 0.3 µM ZN-c3 | 0.3507090072 |
| 0.5 µM triapine + 0 µM ZN-c3 | 0.5 µM triapine + 0.3 µM ZN-c3 | 0.0061193091 |
| 0 µM triapine + 0.3 µM ZN-c3 | 0.5 µM triapine + 0.3 µM ZN-c3 | 0.0266651146 |
| 0.125 µM triapine + 0 µM ZN-c3 | 0.125 µM triapine + 0.4 µM ZN-c3 | 0.0880018341 |
| 0 µM triapine + 0.4 µM ZN-c3 | 0.125 µM triapine + 0.4 µM ZN-c3 | 0.8382434983 |
| 0.25 µM triapine + 0 µM ZN-c3 | 0.25 µM triapine + 0.4 µM ZN-c3 | 0.0196461863 |
| 0 µM triapine + 0.4 µM ZN-c3 | 0.25 µM triapine + 0.4 µM ZN-c3 | 0.2805744595 |
| 0.5 µM triapine + 0 µM ZN-c3 | 0.5 µM triapine + 0.4 µM ZN-c3 | 0.0032247523 |
| 0 µM triapine + 0.4 µM ZN-c3 | 0.5 µM triapine + 0.4 µM ZN-c3 | 0.0441941492 |
| 0.125 µM triapine + 0 µM ZN-c3 | 0.125 µM triapine + 0.5 µM ZN-c3 | 0.0174644168 |
| 0 µM triapine + 0.5 µM ZN-c3 | 0.125 µM triapine + 0.5 µM ZN-c3 | 0.8725688913 |
| 0.25 µM triapine + 0 µM ZN-c3 | 0.25 µM triapine + 0.5 µM ZN-c3 | 0.0058530827 |
| 0 µM triapine + 0.5 µM ZN-c3 | 0.25 µM triapine + 0.5 µM ZN-c3 | 0.4310476894 |
| 0.5 µM triapine + 0 µM ZN-c3 | 0.5 µM triapine + 0.5 µM ZN-c3 | 0.0016349392 |
| 0 µM triapine + 0.5 µM ZN-c3 | 0.5 µM triapine + 0.5 µM ZN-c3 | 0.1331248017 |

Based on data from Fig. 3A, *p* values were calculated with the Kruskal-Wallis test followed by the Dunn's test.

**Additional File 17**

**Table S15. Statistical analysis for triapine-adavosertib-induced caspase 3/7 activity**

WE-68

# Kruskal-Wallis test

| y | n | statistics | df | *p* |
| --- | --- | --- | --- | --- |
| Value | 60 | 52.66604 | 19 | 0.0000526 |

# Dunn's test

| Group 1 | Group 2 | *p* |
| --- | --- | --- |
| 0.125 µM triapine + 0 µM adavosertib | 0.125 µM triapine + 0.1 µM adavosertib | 0.5056830046 |
| 0 µM triapine + 0.1 µM adavosertib | 0.125 µM triapine + 0.1 µM adavosertib | 0.7754554624 |
| 0.25 µM triapine + 0 µM adavosertib | 0.25 µM triapine + 0.1 µM adavosertib | 0.7392948210 |
| 0 µM triapine + 0.1 µM adavosertib | 0.25 µM triapine + 0.1 µM adavosertib | 0.6861399911 |
| 0.5 µM triapine + 0 µM adavosertib | 0.5 µM triapine + 0.1 µM adavosertib | 0.2956057985 |
| 0 µM triapine + 0.1 µM adavosertib | 0.5 µM triapine + 0.1 µM adavosertib | 0.0254548441 |
| 1 µM triapine + 0 µM adavosertib | 1 µM triapine + 0.1 µM adavosertib | 0.1206494940 |
| 0 µM triapine + 0.1 µM adavosertib | 1 µM triapine + 0.1 µM adavosertib | 0.0011277040 |
| 0.125 µM triapine + 0 µM adavosertib | 0.125 µM triapine + 0.2 µM adavosertib | 0.9053921204 |
| 0 µM triapine + 0.2 µM adavosertib | 0.125 µM triapine + 0.2 µM adavosertib | 0.7937256393 |
| 0.25 µM triapine + 0 µM adavosertib | 0.25 µM triapine + 0.2 µM adavosertib | 0.2077278956 |
| 0 µM triapine + 0.2 µM adavosertib | 0.25 µM triapine + 0.2 µM adavosertib | 0.2164335728 |
| 0.5 µM triapine + 0 µM adavosertib | 0.5 µM triapine + 0.2 µM adavosertib | 0.0785743227 |
| 0 µM triapine + 0.2 µM adavosertib | 0.5 µM triapine + 0.2 µM adavosertib | 0.0287506877 |
| 1 µM triapine + 0 µM adavosertib | 1 µM triapine + 0.2 µM adavosertib | 0.1605494020 |
| 0 µM triapine + 0.2 µM adavosertib | 1 µM triapine + 0.2 µM adavosertib | 0.0198319936 |
| 0.125 µM triapine + 0 µM adavosertib | 0.125 µM triapine + 0.5 µM adavosertib | 0.0386366788 |
| 0 µM triapine + 0.5 µM adavosertib | 0.125 µM triapine + 0.5 µM adavosertib | 0.4189767198 |
| 0.25 µM triapine + 0 µM adavosertib | 0.25 µM triapine + 0.5 µM adavosertib | 0.0254548441 |
| 0 µM triapine + 0.5 µM adavosertib | 0.25 µM triapine + 0.5 µM adavosertib | 0.1831398802 |
| 0.5 µM triapine + 0 µM adavosertib | 0.5 µM triapine + 0.5 µM adavosertib | 0.0826968465 |
| 0 µM triapine + 0.5 µM adavosertib | 0.5 µM triapine + 0.5 µM adavosertib | 0.1992790504 |
| 1 µM triapine + 0 µM adavosertib | 1 µM triapine + 0.5 µM adavosertib | 0.2782268668 |
| 0 µM triapine + 0.5 µM adavosertib | 1 µM triapine + 0.5 µM adavosertib | 0.2741988633 |

Based on data from Fig. 2B, *p* values were calculated with the Kruskal-Wallis test followed by the Dunn's test.

SK-ES-1

# Kruskal-Wallis test

| y | n | statistics | df | *p* |
| --- | --- | --- | --- | --- |
| Value | 60 | 57.51568 | 19 | 0.0000095 |

# Dunn's test

| Group 1 | Group 2 | *p* |
| --- | --- | --- |
| 0.125 µM triapine + 0 µM adavosertib | 0.125 µM triap. + 0.05 µM adavosertib | 0.9813491300 |
| 0 µM triapine + 0.05 µM adavosertib | 0.125 µM triap. + 0.05 µM adavosertib | 0.8884504829 |
| 0.25 µM triapine + 0 µM adavosertib | 0.25 µM triapine + 0.05 µM adavosertib | 0.3261707030 |
| 0 µM triapine + 0.05 µM adavosertib | 0.25 µM triapine + 0.05 µM adavosertib | 0.0718496371 |
| 0.5 µM triapine + 0 µM adavosertib | 0.5 µM triapine + 0.05 µM adavosertib | 0.3497361841 |
| 0 µM triapine + 0.05 µM adavosertib | 0.5 µM triapine + 0.05 µM adavosertib | 0.0219635427 |
| 1 µM triapine + 0 µM adavosertib | 1 µM triapine + 0.05 µM adavosertib | 0.5907958141 |
| 0 µM triapine + 0.05 µM adavosertib | 1 µM triapine + 0.05 µM adavosertib | 0.0005892896 |
| 0.125 µM triapine + 0 µM adavosertib | 0.125 µM triapine + 0.1 µM adavosertib | 0.4267089296 |
| 0 µM triapine + 0.1 µM adavosertib | 0.125 µM triapine + 0.1 µM adavosertib | 0.6070383319 |
| 0.25 µM triapine + 0 µM adavosertib | 0.25 µM triapine + 0.1 µM adavosertib | 0.2153421659 |
| 0 µM triapine + 0.1 µM adavosertib | 0.25 µM triapine + 0.1 µM adavosertib | 0.0969537869 |
| 0.5 µM triapine + 0 µM adavosertib | 0.5 µM triapine + 0.1 µM adavosertib | 0.1678102382 |
| 0 µM triapine + 0.1 µM adavosertib | 0.5 µM triapine + 0.1 µM adavosertib | 0.0194000741 |
| 1 µM triapine + 0 µM adavosertib | 1 µM triapine + 0.1 µM adavosertib | 0.4830996864 |
| 0 µM triapine + 0.1 µM adavosertib | 1 µM triapine + 0.1 µM adavosertib | 0.0013613562 |
| 0.125 µM triapine + 0 µM adavosertib | 0.125 µM triapine + 0.2 µM adavosertib | 0.1286267943 |
| 0 µM triapine + 0.2 µM adavosertib | 0.125 µM triapine + 0.2 µM adavosertib | 0.7611980838 |
| 0.25 µM triapine + 0 µM adavosertib | 0.25 µM triapine + 0.2 µM adavosertib | 0.1017502568 |
| 0 µM triapine + 0.2 µM adavosertib | 0.25 µM triapine + 0.2 µM adavosertib | 0.2618108446 |
| 0.5 µM triapine + 0 µM adavosertib | 0.5 µM triapine + 0.2 µM adavosertib | 0.0836415155 |
| 0 µM triapine + 0.2 µM adavosertib | 0.5 µM triapine + 0.2 µM adavosertib | 0.0795480174 |
| 1 µM triapine + 0 µM adavosertib | 1 µM triapine + 0.2 µM adavosertib | 0.5279141664 |
| 0 µM triapine + 0.2 µM adavosertib | 1 µM triapine + 0.2 µM adavosertib | 0.0279856371 |

Based on data from Fig. 2B, *p* values were calculated with the Kruskal-Wallis test followed by the Dunn's test.

A673

# Kruskal-Wallis test

| y | n | statistics | df | *p* |
| --- | --- | --- | --- | --- |
| Value | 60 | 53.89342 | 19 | 0.0000343 |

# Dunn's test

| Group 1 | Group 2 | *p* |
| --- | --- | --- |
| 0.125 µM triapine + 0 µM adavosertib | 0.125 µM triapine + 0.1 µM adavosertib | 0.5907958141 |
| 0 µM triapine + 0.1 µM adavosertib | 0.125 µM triapine + 0.1 µM adavosertib | 0.5589255280 |
| 0.25 µM triapine + 0 µM adavosertib | 0.25 µM triapine + 0.1 µM adavosertib | 0.4000169190 |
| 0 µM triapine + 0.1 µM adavosertib | 0.25 µM triapine + 0.1 µM adavosertib | 0.6739044116 |
| 0.5 µM triapine + 0 µM adavosertib | 0.5 µM triapine + 0.1 µM adavosertib | 0.0718496371 |
| 0 µM triapine + 0.1 µM adavosertib | 0.5 µM triapine + 0.1 µM adavosertib | 0.7434520373 |
| 1 µM triapine + 0 µM adavosertib | 1 µM triapine + 0.1 µM adavosertib | 0.1228509483 |
| 0 µM triapine + 0.1 µM adavosertib | 1 µM triapine + 0.1 µM adavosertib | 0.1119087760 |
| 0.125 µM triapine + 0 µM adavosertib | 0.125 µM triapine + 0.2 µM adavosertib | 0.1346114943 |
| 0 µM triapine + 0.2 µM adavosertib | 0.125 µM triapine + 0.2 µM adavosertib | 0.9440881316 |
| 0.25 µM triapine + 0 µM adavosertib | 0.25 µM triapine + 0.2 µM adavosertib | 0.0836415155 |
| 0 µM triapine + 0.2 µM adavosertib | 0.25 µM triapine + 0.2 µM adavosertib | 0.8700124298 |
| 0.5 µM triapine + 0 µM adavosertib | 0.5 µM triapine + 0.2 µM adavosertib | 0.0066920313 |
| 0 µM triapine + 0.2 µM adavosertib | 0.5 µM triapine + 0.2 µM adavosertib | 0.3497361841 |
| 1 µM triapine + 0 µM adavosertib | 1 µM triapine + 0.2 µM adavosertib | 0.0552433544 |
| 0 µM triapine + 0.2 µM adavosertib | 1 µM triapine + 0.2 µM adavosertib | 0.0969537869 |
| 0.125 µM triapine + 0 µM adavosertib | 0.125 µM triapine + 0.5 µM adavosertib | 0.0029882302 |
| 0 µM triapine + 0.5 µM adavosertib | 0.125 µM triapine + 0.5 µM adavosertib | 0.9813491300 |
| 0.25 µM triapine + 0 µM adavosertib | 0.25 µM triapine + 0.5 µM adavosertib | 0.0021952860 |
| 0 µM triapine + 0.5 µM adavosertib | 0.25 µM triapine + 0.5 µM adavosertib | 0.9440881316 |
| 0.5 µM triapine + 0 µM adavosertib | 0.5 µM triapine + 0.5 µM adavosertib | 0.0004155490 |
| 0 µM triapine + 0.5 µM adavosertib | 0.5 µM triapine + 0.5 µM adavosertib | 0.8516447746 |
| 1 µM triapine + 0 µM adavosertib | 1 µM triapine + 0.5 µM adavosertib | 0.0879039466 |
| 0 µM triapine + 0.5 µM adavosertib | 1 µM triapine + 0.5 µM adavosertib | 0.9069490918 |

Based on data from Fig. 2B, *p* values were calculated with the Kruskal-Wallis test followed by the Dunn's test.

**Additional File 18**

**Table S16. Statistical analysis for triapine-ZN-c3-induced caspase 3/7 activity**

WE-68

# Kruskal-Wallis test

| y | n | statistics | df | *p* |
| --- | --- | --- | --- | --- |
| Value | 51 | 44.23409 | 15 | 0.000101 |

# Dunn's test

| Group 1 | Group 2 | *p* |
| --- | --- | --- |
| 0.125 µM triapine + 0 µM ZN-c3 | 0.125 µM triapine + 0.3 µM ZN-c3 | 0.7263648909 |
| 0 µM triapine + 0.3 µM ZN-c3 | 0.125 µM triapine + 0.3 µM ZN-c3 | 0.5402473968 |
| 0.25 µM triapine + 0 µM ZN-c3 | 0.25 µM triapine + 0.3 µM ZN-c3 | 0.0055960500 |
| 0 µM triapine + 0.3 µM ZN-c3 | 0.25 µM triapine + 0.3 µM ZN-c3 | 0.0051146826 |
| 0.5 µM triapine + 0 µM ZN-c3 | 0.5 µM triapine + 0.3 µM ZN-c3 | 0.0542533777 |
| 0 µM triapine + 0.3 µM ZN-c3 | 0.5 µM triapine + 0.3 µM ZN-c3 | 0.0019924301 |
| 0.125 µM triapine + 0 µM ZN-c3 | 0.125 µM triapine + 0.4 µM ZN-c3 | 0.1152927016 |
| 0 µM triapine + 0.4 µM ZN-c3 | 0.125 µM triapine + 0.4 µM ZN-c3 | 0.5597080791 |
| 0.25 µM triapine + 0 µM ZN-c3 | 0.25 µM triapine + 0.4 µM ZN-c3 | 0.0007970068 |
| 0 µM triapine + 0.4 µM ZN-c3 | 0.25 µM triapine + 0.4 µM ZN-c3 | 0.0332580376 |
| 0.5 µM triapine + 0 µM ZN-c3 | 0.5 µM triapine + 0.4 µM ZN-c3 | 0.0661631462 |
| 0 µM triapine + 0.4 µM ZN-c3 | 0.5 µM triapine + 0.4 µM ZN-c3 | 0.0801484306 |
| 0.125 µM triapine + 0 µM ZN-c3 | 0.125 µM triapine + 0.5 µM ZN-c3 | 0.0507045524 |
| 0 µM triapine + 0.5 µM ZN-c3 | 0.125 µM triapine + 0.5 µM ZN-c3 | 0.5597080791 |
| 0.25 µM triapine + 0 µM ZN-c3 | 0.25 µM triapine + 0.5 µM ZN-c3 | 0.0009824507 |
| 0 µM triapine + 0.5 µM ZN-c3 | 0.25 µM triapine + 0.5 µM ZN-c3 | 0.0907417342 |
| 0.5 µM triapine + 0 µM ZN-c3 | 0.5 µM triapine + 0.5 µM ZN-c3 | 0.1529972920 |
| 0 µM triapine + 0.5 µM ZN-c3 | 0.5 µM triapine + 0.5 µM ZN-c3 | 0.3358466950 |

Based on data from Fig. 3B, *p* values were calculated with the Kruskal-Wallis test followed by the Dunn's test.

SK-ES-1

# Kruskal-Wallis test

| y | n | statistics | df | *p* |
| --- | --- | --- | --- | --- |
| Value | 51 | 45.17647 | 15 | 0.0000718 |

# Dunn's test

| Group 1 | Group 2 | *p* |
| --- | --- | --- |
| 0.125 µM triapine + 0 µM ZN-c3 | 0.125 µM triapine + 0.3 µM ZN-c3 | 0.2937662551 |
| 0 µM triapine + 0.3 µM ZN-c3 | 0.125 µM triapine + 0.3 µM ZN-c3 | 0.7929561637 |
| 0.25 µM triapine + 0 µM ZN-c3 | 0.25 µM triapine + 0.3 µM ZN-c3 | 0.0212261861 |
| 0 µM triapine + 0.3 µM ZN-c3 | 0.25 µM triapine + 0.3 µM ZN-c3 | 0.1221820669 |
| 0.5 µM triapine + 0 µM ZN-c3 | 0.5 µM triapine + 0.3 µM ZN-c3 | 0.0051146826 |
| 0 µM triapine + 0.3 µM ZN-c3 | 0.5 µM triapine + 0.3 µM ZN-c3 | 0.0102757074 |
| 0.125 µM triapine + 0 µM ZN-c3 | 0.125 µM triapine + 0.4 µM ZN-c3 | 0.1024331512 |
| 0 µM triapine + 0.4 µM ZN-c3 | 0.125 µM triapine + 0.4 µM ZN-c3 | 0.7705637377 |
| 0.25 µM triapine + 0 µM ZN-c3 | 0.25 µM triapine + 0.4 µM ZN-c3 | 0.0079566933 |
| 0 µM triapine + 0.4 µM ZN-c3 | 0.25 µM triapine + 0.4 µM ZN-c3 | 0.1797475028 |
| 0.5 µM triapine + 0 µM ZN-c3 | 0.5 µM triapine + 0.4 µM ZN-c3 | 0.0055960500 |
| 0 µM triapine + 0.4 µM ZN-c3 | 0.5 µM triapine + 0.4 µM ZN-c3 | 0.0473522898 |
| 0.125 µM triapine + 0 µM ZN-c3 | 0.125 µM triapine + 0.5 µM ZN-c3 | 0.0196428631 |
| 0 µM triapine + 0.5 µM ZN-c3 | 0.125 µM triapine + 0.5 µM ZN-c3 | 0.6200468927 |
| 0.25 µM triapine + 0 µM ZN-c3 | 0.25 µM triapine + 0.5 µM ZN-c3 | 0.0032239190 |
| 0 µM triapine + 0.5 µM ZN-c3 | 0.25 µM triapine + 0.5 µM ZN-c3 | 0.2553767193 |
| 0.5 µM triapine + 0 µM ZN-c3 | 0.5 µM triapine + 0.5 µM ZN-c3 | 0.0042625550 |
| 0 µM triapine + 0.5 µM ZN-c3 | 0.5 µM triapine + 0.5 µM ZN-c3 | 0.1152927016 |

Based on data from Fig. 3B, *p* values were calculated with the Kruskal-Wallis test followed by the Dunn's test.

A673

# Kruskal-Wallis test

| y | n | statistics | df | *p* |
| --- | --- | --- | --- | --- |
| Value | 51 | 34.25233 | 15 | 0.00314 |

# Dunn's test

| Group 1 | Group 2 | *p* |
| --- | --- | --- |
| 0.125 µM triapine + 0 µM ZN-c3 | 0.125 µM triapine + 0.3 µM ZN-c3 | 0.2677656016 |
| 0 µM triapine + 0.3 µM ZN-c3 | 0.125 µM triapine + 0.3 µM ZN-c3 | 0.8382391682 |
| 0.25 µM triapine + 0 µM ZN-c3 | 0.25 µM triapine + 0.3 µM ZN-c3 | 0.1797475028 |
| 0 µM triapine + 0.3 µM ZN-c3 | 0.25 µM triapine + 0.3 µM ZN-c3 | 1.0000000000 |
| 0.5 µM triapine + 0 µM ZN-c3 | 0.5 µM triapine + 0.3 µM ZN-c3 | 0.0121388080 |
| 0 µM triapine + 0.3 µM ZN-c3 | 0.5 µM triapine + 0.3 µM ZN-c3 | 0.4310351818 |
| 0.125 µM triapine + 0 µM ZN-c3 | 0.125 µM triapine + 0.4 µM ZN-c3 | 0.0181636819 |
| 0 µM triapine + 0.4 µM ZN-c3 | 0.125 µM triapine + 0.4 µM ZN-c3 | 0.8610933458 |
| 0.25 µM triapine + 0 µM ZN-c3 | 0.25 µM triapine + 0.4 µM ZN-c3 | 0.0332580376 |
| 0 µM triapine + 0.4 µM ZN-c3 | 0.25 µM triapine + 0.4 µM ZN-c3 | 0.9302810207 |
| 0.5 µM triapine + 0 µM ZN-c3 | 0.5 µM triapine + 0.4 µM ZN-c3 | 0.0079566933 |
| 0 µM triapine + 0.4 µM ZN-c3 | 0.5 µM triapine + 0.4 µM ZN-c3 | 0.9534877499 |
| 0.125 µM triapine + 0 µM ZN-c3 | 0.125 µM triapine + 0.5 µM ZN-c3 | 0.0061179233 |
| 0 µM triapine + 0.5 µM ZN-c3 | 0.125 µM triapine + 0.5 µM ZN-c3 | 0.8155206431 |
| 0.25 µM triapine + 0 µM ZN-c3 | 0.25 µM triapine + 0.5 µM ZN-c3 | 0.0154952608 |
| 0 µM triapine + 0.5 µM ZN-c3 | 0.25 µM triapine + 0.5 µM ZN-c3 | 0.5597080791 |
| 0.5 µM triapine + 0 µM ZN-c3 | 0.5 µM triapine + 0.5 µM ZN-c3 | 0.0094432934 |
| 0 µM triapine + 0.5 µM ZN-c3 | 0.5 µM triapine + 0.5 µM ZN-c3 | 0.4310351818 |

Based on data from Fig. 3B, *p* values were calculated with the Kruskal-Wallis test followed by the Dunn's test.
