# Supplementary material for: Combined inhibition of ribonucleotide reductase and WEE1 induces synergistic anticancer activity in Ewing’s sarcoma cells
Source: BMC Cancer. 2025 Feb 17;25:277. doi: 10.1186/s12885-025-13691-2 (PMC11831844; doi:10.1186/s12885-025-13691-2)
Supplement: Supplementary file 2 — Additional file S7: Figure S1: Effects of PARP inhibitors and adavosertib in Ewing’s sarcoma cells. Cells were exposed to drugs for 48 h. (A) Cell death and (B) Δψm loss were determined by flow-cytometric analysis of propidium iodide uptake and DiOC6(3) staining, respectively. Means ± SEM of each three independent measurements are shown. Additional file S8: Figure S2: RNRi and WEE1i do not affect TP53 expression. Cells were exposed to triapine in combination with adavosertib or ZN-c3 for 24 h. TP53 expression levels were determined by real-time RT-PCR and normalised to B2M expression levels; relative gene expression levels are the ratio of treated cells to untreated cells. Means ± SEM of each three independent measurements are shown [file 12885_2025_13691_MOESM2_ESM.pptx]

## Slide 1
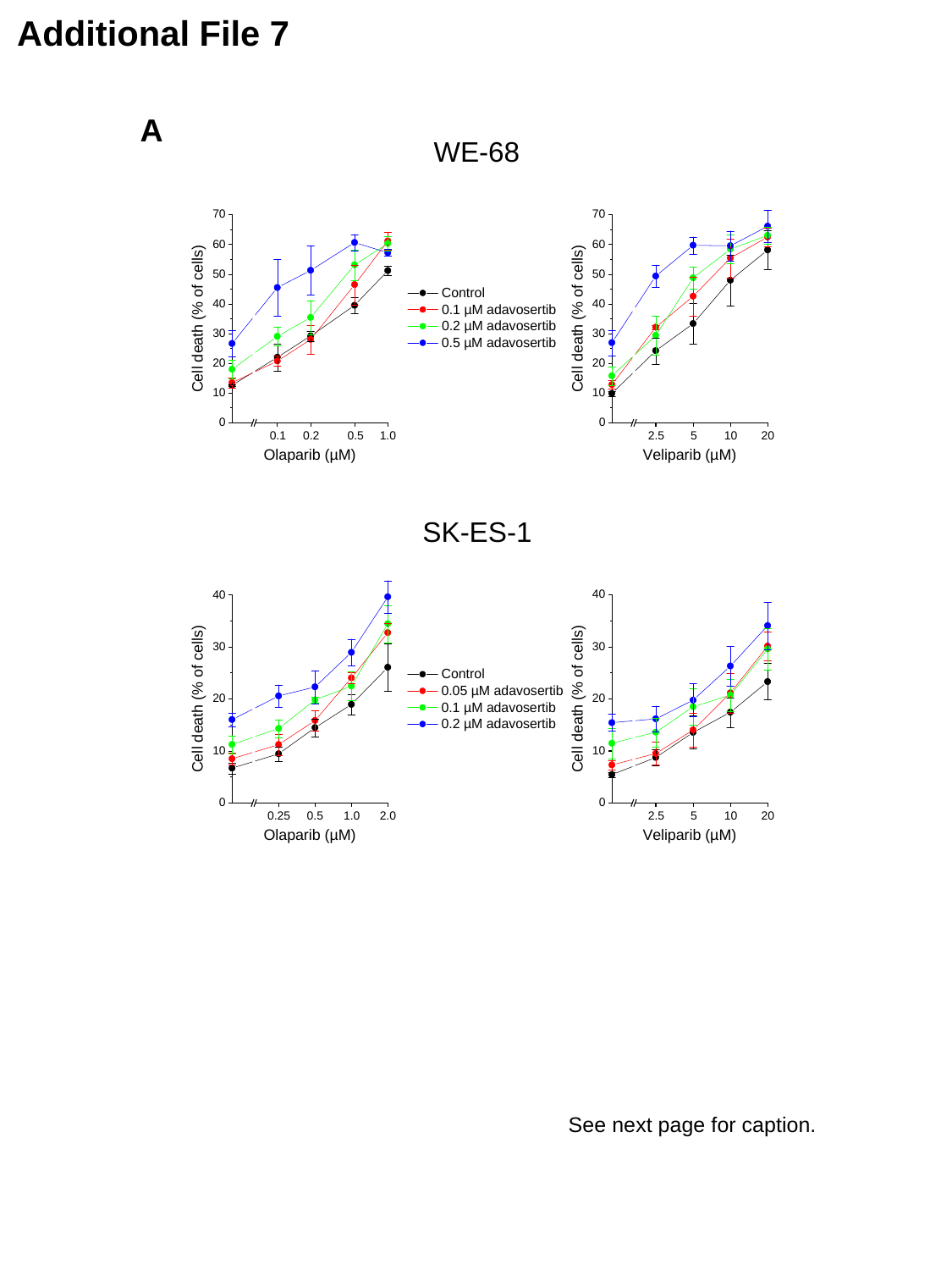

Additional File 7
A
WE-68
SK-ES-1
See next page for caption.

## Slide 2
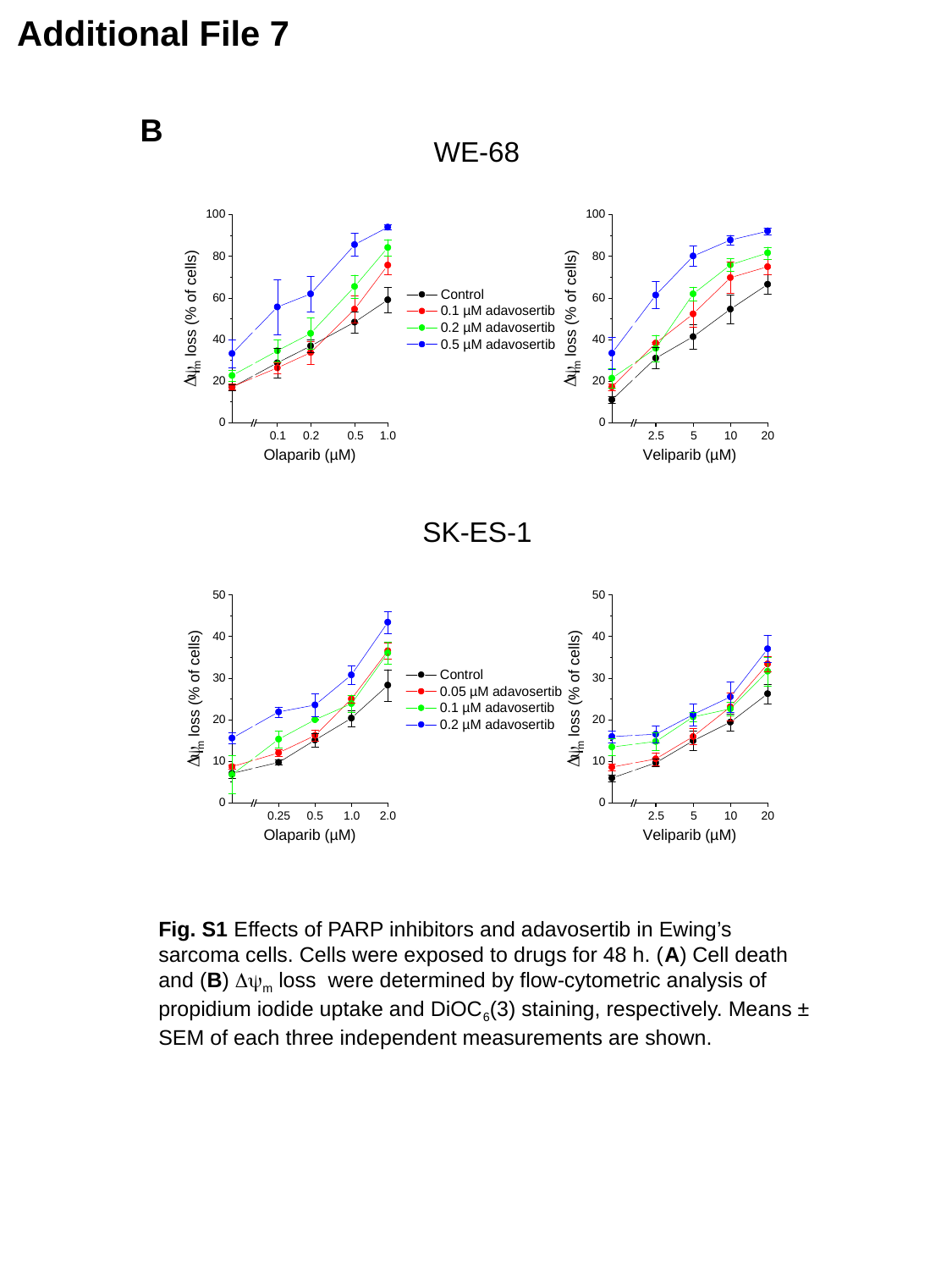

Additional File 7
B
WE-68
SK-ES-1
Fig. S1 Effects of PARP inhibitors and adavosertib in Ewing’s sarcoma cells. Cells were exposed to drugs for 48 h. (A) Cell death and (B) Dym loss were determined by flow-cytometric analysis of propidium iodide uptake and DiOC6(3) staining, respectively. Means ± SEM of each three independent measurements are shown.

## Slide 3
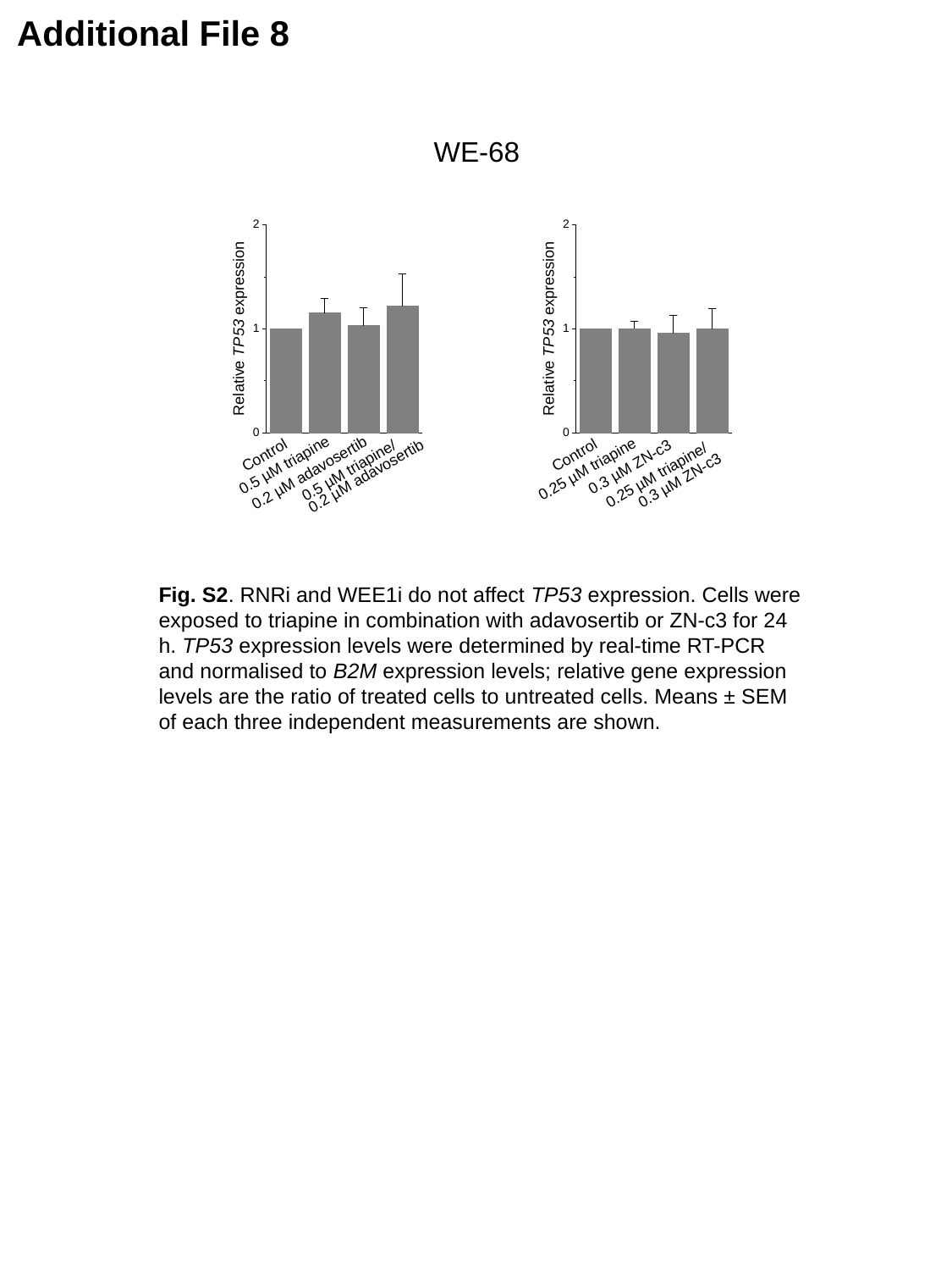

Additional File 8
WE-68
Fig. S2. RNRi and WEE1i do not affect TP53 expression. Cells were exposed to triapine in combination with adavosertib or ZN-c3 for 24 h. TP53 expression levels were determined by real-time RT-PCR and normalised to B2M expression levels; relative gene expression levels are the ratio of treated cells to untreated cells. Means ± SEM of each three independent measurements are shown.
