# Supplementary material for: Combined inhibition of ribonucleotide reductase and WEE1 induces synergistic anticancer activity in Ewing’s sarcoma cells
Source: BMC Cancer. 2025 Feb 17;25:277. doi: 10.1186/s12885-025-13691-2 (PMC11831844; doi:10.1186/s12885-025-13691-2)
Supplement: Supplementary file 3 — Supplementary Material 3 [file 12885_2025_13691_MOESM3_ESM.pptx]

## Slide 1
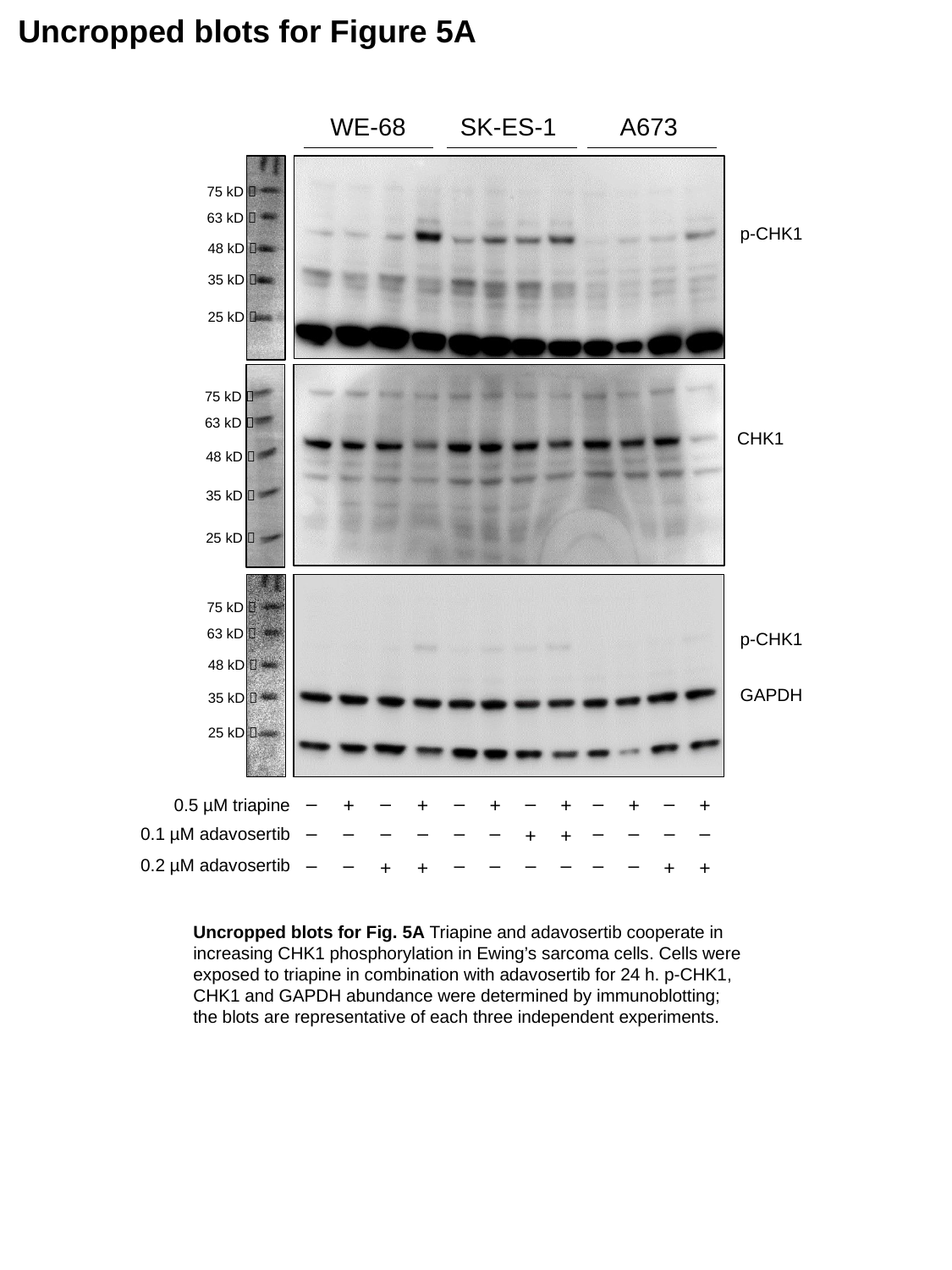

Uncropped blots for Figure 5A
WE-68
SK-ES-1
A673
75 kD 
63 kD 
 48 kD 
 35 kD 
 25 kD 
p-CHK1
75 kD 
63 kD 
CHK1
 48 kD 
 35 kD 
 25 kD 
75 kD 
63 kD 
 48 kD 
 35 kD 
 25 kD 
p-CHK1
GAPDH
–
–
–
–
–
–
+
+
+
+
+
+
0.5 µM triapine
–
–
–
–
–
–
–
–
–
–
0.1 µM adavosertib
+
+
–
–
–
–
–
–
–
–
0.2 µM adavosertib
+
+
+
+
Uncropped blots for Fig. 5A Triapine and adavosertib cooperate in increasing CHK1 phosphorylation in Ewing’s sarcoma cells. Cells were exposed to triapine in combination with adavosertib for 24 h. p-CHK1, CHK1 and GAPDH abundance were determined by immunoblotting; the blots are representative of each three independent experiments.

## Slide 2
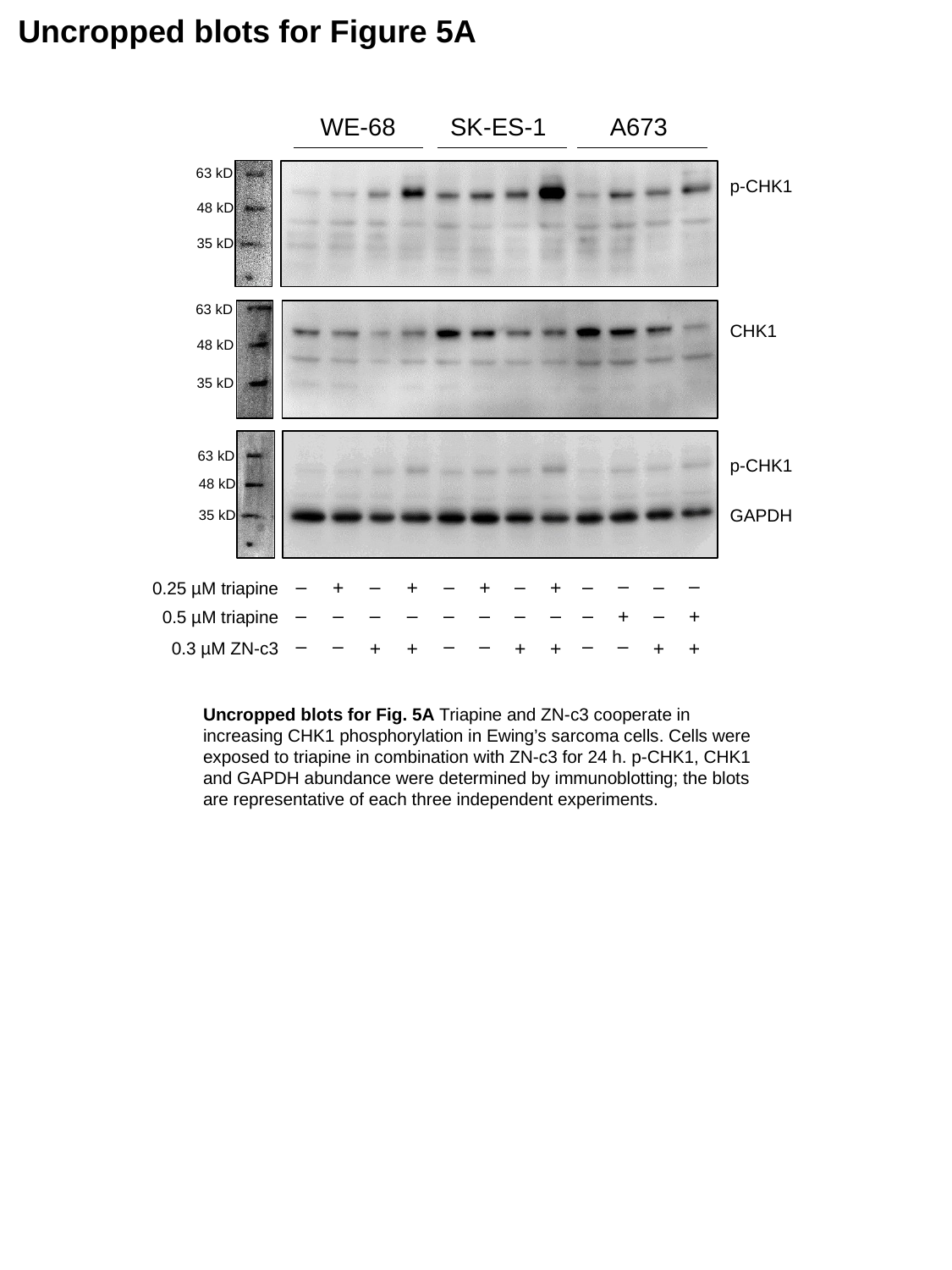

Uncropped blots for Figure 5A
WE-68
SK-ES-1
A673
63 kD 
 48 kD 
 35 kD 
p-CHK1
63 kD 
 48 kD 
 35 kD 
CHK1
63 kD 
 48 kD 
 35 kD 
p-CHK1
GAPDH
–
–
–
–
–
–
–
–
+
+
+
+
0.25 µM triapine
–
–
–
–
–
–
–
–
–
–
+
+
0.5 µM triapine
–
–
–
–
–
–
0.3 µM ZN-c3
+
+
+
+
+
+
Uncropped blots for Fig. 5A Triapine and ZN-c3 cooperate in increasing CHK1 phosphorylation in Ewing’s sarcoma cells. Cells were exposed to triapine in combination with ZN-c3 for 24 h. p-CHK1, CHK1 and GAPDH abundance were determined by immunoblotting; the blots are representative of each three independent experiments.

## Slide 3
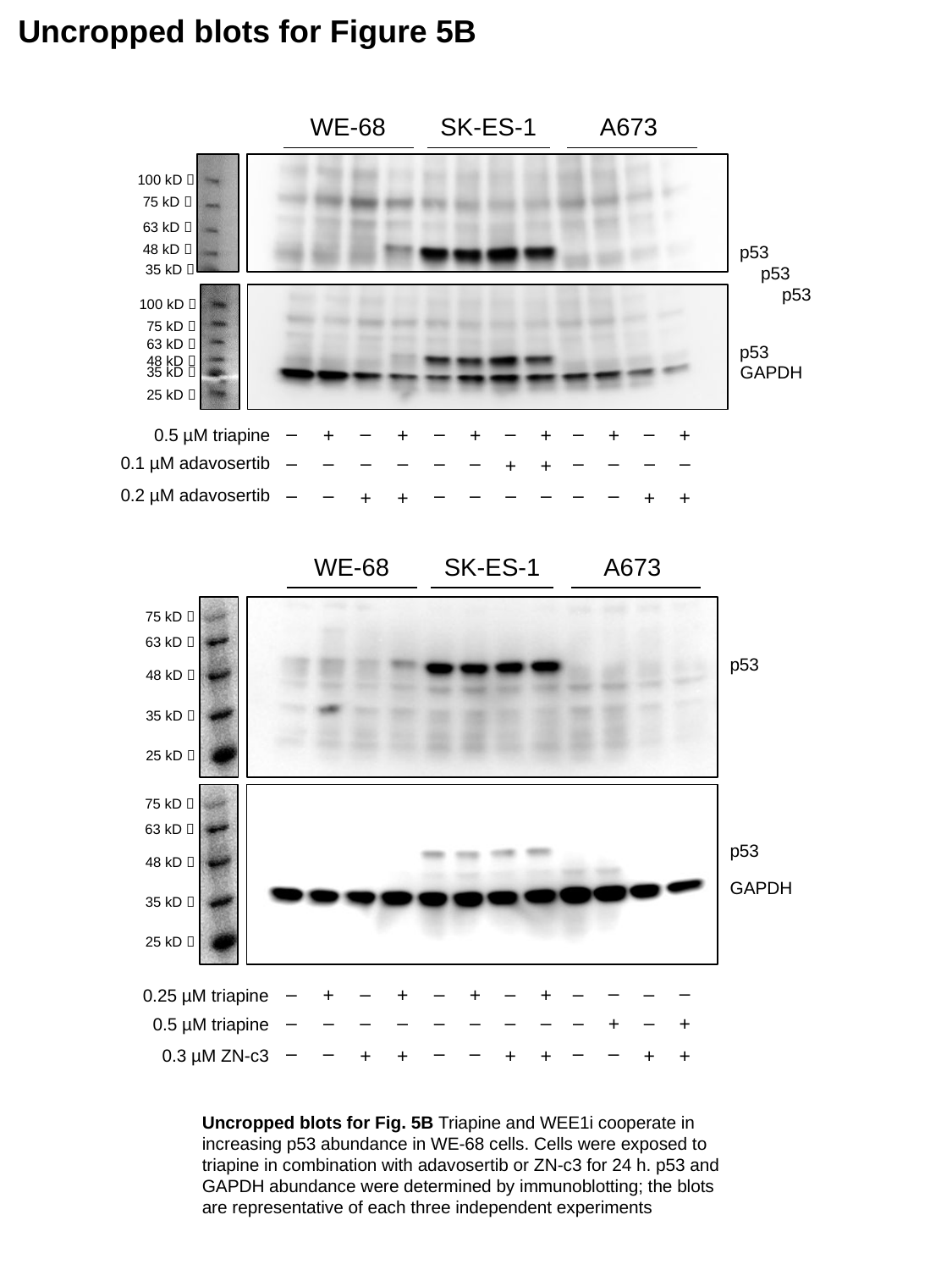

Uncropped blots for Figure 5B
WE-68
SK-ES-1
A673
75 kD 
63 kD 
48 kD 
100 kD 
p53
35 kD 
100 kD 
75 kD 
63 kD 
48 kD 
35 kD 
25 kD 
p53
GAPDH
–
–
–
–
–
–
+
+
+
+
+
+
0.5 µM triapine
–
–
–
–
–
–
–
–
–
–
0.1 µM adavosertib
+
+
–
–
–
–
–
–
–
–
0.2 µM adavosertib
+
+
+
+
WE-68
SK-ES-1
A673
75 kD 
63 kD 
 48 kD 
 35 kD 
 25 kD 
75 kD 
63 kD 
 48 kD 
 35 kD 
 25 kD 
p53
GAPDH
–
–
–
–
–
–
–
–
+
+
+
+
0.25 µM triapine
–
–
–
–
–
–
–
–
–
–
+
+
0.5 µM triapine
–
–
–
–
–
–
0.3 µM ZN-c3
+
+
+
+
+
+
p53
p53
p53
Uncropped blots for Fig. 5B Triapine and WEE1i cooperate in increasing p53 abundance in WE-68 cells. Cells were exposed to triapine in combination with adavosertib or ZN-c3 for 24 h. p53 and GAPDH abundance were determined by immunoblotting; the blots are representative of each three independent experiments
